# Coating Metal–Organic Frameworks (MOFs) and Associated Composites on Electrodes, Thin Film Polymeric Materials, and Glass Surfaces

**DOI:** 10.3390/nano15151187

**Published:** 2025-08-02

**Authors:** Md Zahidul Hasan, Tyeaba Tasnim Dipti, Liu Liu, Caixia Wan, Li Feng, Zhongyu Yang

**Affiliations:** 1 Department of Chemical and Biomedical Engineering, University of Missouri-Columbia, Columbia, MO 65211, USA; mhdbk@missouri.edu (M.Z.H.); td6cp@missouri.edu (T.T.D.); lltkw@missouri.edu (L.L.); wanca@missouri.edu (C.W.); 2 Materials Science & Engineering Institute, University of Missouri, Columbia, MO 65211, USA

**Keywords:** Metal–Organic Frameworks, MOF coating, electrode, polymers, glass surfaces

## Abstract

Metal–Organic Frameworks (MOFs) have emerged as advanced porous crystalline materials due to their highly ordered structures, ultra-high surface areas, fine-tunable pore sizes, and massive chemical diversity. These features, arising from the coordination between an almost unlimited number of metal ions/clusters and organic linkers, have resulted in significant interest in MOFs for applications in gas storage, catalysis, sensing, energy, and biomedicine. Beyond their stand-alone properties and applications, recent research has increasingly explored the integration of MOFs with other substrates, particularly electrodes, polymeric thin films, and glass surfaces, to create synergistic effects that enhance material performance and broaden application potential. Coating MOFs onto these substrates can yield significant benefits, including, but not limited to, improved sensitivity and selectivity in electrochemical sensors, enhanced mechanical and separation properties in membranes, and multifunctional coatings for optical and environmental applications. This review provides a comprehensive and up-to-date summary of recent advances (primarily from the past 3–5 years) in MOF coating techniques, including layer-by-layer assembly, in situ growth, and electrochemical deposition. This is followed by a discussion of the representative applications arising from MOF-substrate coating and an outline of key challenges and future directions in this rapidly evolving field. This article aims to serve as a focused reference point for researchers interested in both fundamental strategies and applied developments in MOF surface coatings.

## 1. Introduction

Metal–Organic Frameworks (MOFs), a unique class of porous crystalline materials, are composed of extensive coordination bonds among building blocks of metal ions or metal clusters and organic ligands. This unique feature endorses MOFs highly ordered, uniform, and porous structures with extremely high surface areas, tunable pore sizes, and diverse functionalities [1,2,3]. Typical MOF topologies usually span a broad range of sizes and shapes across 1D to 3D dimensionalities. In principle, there is an unlimited number of MOFs based on the varied combinations of metal ion–ligand, given the high diversity of metal ions/clusters and organic ligands, resulting in not only a huge number of MOF structures published nowadays but unlimited possibilities of new materials design with tailored functions or properties. The typical applications of MOFs include, but are not limited to, gas adsorption and separation, chemical catalysis, sensing, storage, energy, therapeutics, enzyme immobilization, cell-free biocatalysis, wastewater treatment, and drug delivery [4,5,6,7,8,9,10,11,12].

Although most MOF applications to date have focused on MOFs themselves, integrating MOFs with other materials, such as electrodes, polymers, and glass surfaces, has begun to emerge, which often endorses these materials enhanced/advanced performances due to the synergistic effects [13,14,15,16,17,18,19,20,21]. For example, coating MOFs onto electrode surfaces can significantly improve the sensitivity and selectivity of electrochemical sensors as well as enhance capacities as battery and supercapacitor materials, opening new avenues for energy conversion and electrocatalysis [17,22]. Incorporating MOFs into thin-film polymeric materials, on the other hand, can not only improve the mechanical, thermal, and chemical properties of the thin films but provide an opportunity to promote separation and/or storage capabilities by forming functional membranes [23]. The deposition of MOFs on glass surfaces can allow for the fabrication of functional coatings with applications in optics, catalysis, and environment protection. As more efforts and attention are directed in these areas, one would only anticipate an increase in the integration of MOFs with electrodes, polymers, and glass surfaces. Although review articles focused on MOF coatings or on coating of MOFs on different material surfaces exist, there is a need for an up-to-date summary of MOF coatings on three surfaces, including electrodes, polymers, and glass.

This review aims to summarize the recent advances in coating MOFs onto electrodes, thin-film polymeric materials, and glass surfaces. Our discussion will start with various commonly used methods for coating MOFs on different surfaces, such as layer-by-layer assembly, in situ growth, and electrochemical deposition. We will also briefly highlight the potential applications of coating MOFs onto various surfaces in several fields. Finally, this review will conclude with a summary of the challenges and future directions in this rapidly emerging field. Due to the massive amount of relevant literature, we will only highlight the most typical representation of a technique or application, ideally within the past 3–5 years. Interested readers are referred to the literature cited through this article. A comprehensive summary of the involved coating methods and the key factors to consider is presented in Table 1.

## 2. Approaches and Strategies for Coating MOFs on Various Surfaces

### 2.1. Electrochemical Deposition

**Figure 1:**
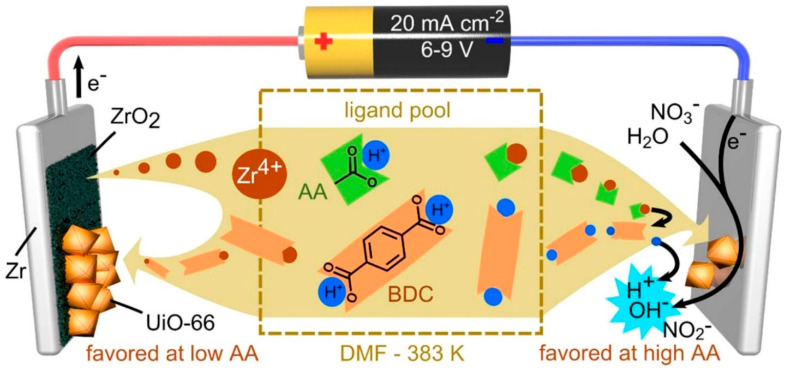
Representative scheme of the anodic and cathodic electrochemical deposition mechanisms of MOFs [24]. Copyright 2015, American Chemical Society.

Electrochemical deposition is a classic technique that utilizes controlled current or electric potential to deposit materials from a solution onto (often conductive) surfaces via electrochemical (redox) reactions. It is widely used in electroplating, battery fabrication, and thin-film materials development. Electrochemical deposition has also been applied to MOF synthesis recently to allow for the controlled growth of MOFs on specific surfaces, to offer tunable thickness and morphology, and to ensure compatibility with electronic devices for sensor, battery, and supercapacitor applications. Depending on the working mechanisms, electrochemical deposition can be categorized into anodic electrodeposition, cathodic electrodeposition, and electrophoretic deposition (EPD), with the former two more commonly seen in the MOF-deposition literature (Figure 1) [24].

Anodic electrodeposition differs from cathodic electrodeposition in many ways. For example, anodic electrodeposition relies on an oxidation reaction at the anode of an electrochemical cell, wherein negatively charged species are attracted to the positively charged electrode while the anode metal is oxidized and becomes M^n+^, which dissolves into the solution. Such a release of metal ions triggers the formation of MOFs when ligands are present in the same solution. The typical voltage required to achieve this process depends on the metal ions. The technique can be limited by the anode materials, which must be conductive and are often sacrificial upon MOF formation. Nevertheless, several MOFs, such as Cu-BTC, Ce-MOFs, Ni-MOF-74, and HKUST-1 MOF, as reported in refs. [25,26,27,28,29,30,31], have been electrochemically synthesized and deposited onto electrode/metal surfaces by anodic electrodeposition. Cathodic electrodeposition happens at the cathode of an electrochemical cell wherein positively charged species are attracted to the electrode surface while water or NO_3_^−^ is reduced to generate OH^−^. Such an increase in local pH triggers the deprotonation of typical ligands, such as H-BDC, generating BDC^−^. The deprotonated ligand then reacts with the metal ions in the solution to synthesize MOFs. It is more convenient to control the composition of the formed MOFs as the metal ions can be controlled in the solution. The surface is also not limited to conductive electrodes; inert polymeric thin films and non-conductive glass can also be deposited with the formed MOFs. Because of this advantage, many MOFs, such as ZIF-8, ZIF-67, Co-MOF, Fe/Co-MOF, MOF-5, and MIL-100, have been synthesized with cathodic electrodeposition [32,33,34,35,36,37,38,39]. The typical voltage applied to achieve this process is ~−1.5 V.

**Figure 2:**
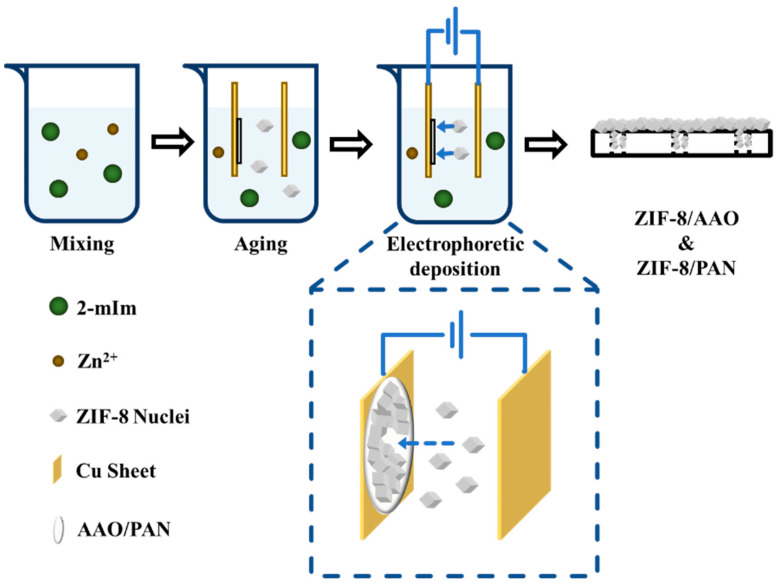
Representative scheme of the preparation process of ZIF-8 MOFs membrane via the electrophoretic deposition method. Reproduced with permission from Ref. [40]. Open access. MDPI.

A third way to perform electrochemical deposition is through EPD (Figure 2), as discussed in ref. [40], in which the often positively charged MOFs, such as UiO-66, UiO-66-NH_2_, and Ni-MOF-74 [26], can be immobilized on the negatively charged, fluoride-doped tin oxide glass, with UiO-66 and UiO-66-NH_2_ films possessing high stability in basic oxidative aqueous solutions. In comparison to anodic and cathodic electrochemical deposition, EPD has the advantage of simpler operation and is more universal; anodic and cathodic electrochemical deposition, on the other hand, offer more control in current or potential and, thus, possible morphology, thickness, and crystallinity. The stability of the MOF films formed through EPD, however, may require additional design to meet the desired level.

### 2.2. In Situ (Solvothermal/Hydrothermal) Growth

Solvothermal/hydrothermal in situ growth of MOFs is the most common means to prepare MOFs, offering advantages, such as controlled morphology and orientation, high crystallinity and purity, as well as high yield, scalability, and reproducibility. The in situ growth of MOFs on surfaces, like metal, glass, or polymer thin films, via solvothermal or hydrothermal methods is also widely used for coating applications in sensing, catalysis, and separation, among other applications. In comparison to electrochemical deposition, solvothermal/hydrothermal in situ growth offers stronger adhesion, more uniform coatings, and better integration with substrate devices (membranes, sensors, electrodes, etc.).

The principle is quite straightforward: mixing metal ions and ligands together with the substrate surface (glass, metal, or polymeric thin films) in organic solvents, such as DMF or ethanol (solvothermal), or in water (hydrothermal), at elevated temperatures and pressures. MOFs then grow directly on the surfaces of the substrates that are immersed in the solution. Surface cleaning is usually critical to avoid contamination and defects. In addition to the regular DI water wash, sonication, 1 M HCl, and organic solvent are also applied in cleaning to remove organic matter and the oxide layer. Pretreatment of the cleaned surfaces is often required through oxygen plasma, acid/base etching, silanization, or self-assembled monolayers to improve surface hydrophilicity, to include anchoring sites for metal ions, or to create a desired charge distribution. These factors usually determine nucleation density and adhesion. Then, metal ions in solution interact with the key functional groups on the surface (usually –OH, –COOH, –NH_2_) as crystallization nuclei, followed by coordination with the first layer of ligands and the next layers. It is also possible to have island growth depending on surface energy, concentrations of metal ions and ligands (which affect crystallinity and phase purity), ligand diffusion efficiency, and reaction temperature (a higher temperature usually leads to faster nucleation)/duration (the longer the reaction, the thicker the films). Uniform thin MOF films, vertically oriented crystals, and nano-/micro-structured coatings can usually form. It is also common to include modulators to control crystallization speed and defect formation.

**Figure 3:**
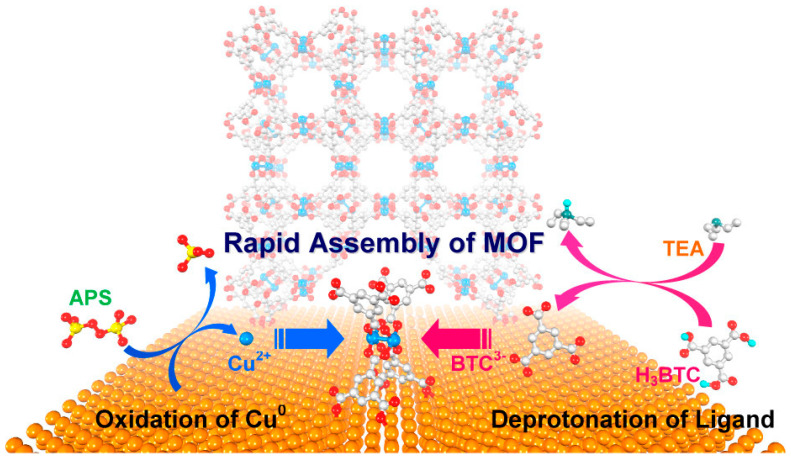
Representative scheme of the direct in situ conversion of Cu metal into HKUST-1. The left and right sides of the scheme represent the oxidation of metallic Cu by APS and the deprotonation of the H_3_BTC ligand by TEA, respectively. (Atom colors: yellow = sulfur; red = oxygen; grey = carbon; cyan = hydrogen; green = nitrogen; blue = Cu.) Reproduced with permission from Ref. [42]. Copyright 2016, American Chemical Society.

Typical examples of in situ (solvothermal/hydrothermal) growth of MOFs are mostly on metal surfaces (Figure 3), such as Cu-BTC on Cu foil and Ni-MOF on Ni foam, as shown in ref. [41], likely due to the low thermal stability of glass and polymeric thin films. In one case [42], a cleaned grey Ni foam (NF) was treated in thiourea/water and Ni(NO_3_)/DMF under elevated temperature (60–120 °C) sequentially to generate Ni_3_S_2_ and MOF-derived Ni(OH)_2_ composite electrode materials on NF, which showed exceptional supercapacitor performances. In another work [43], the temperature of solvothermal growth of Ni-MOFs on NF surfaces was found to impact the morphology and supercapacitor performance of the coating, suggesting an intriguing way to tune the functionality of the MOF-coated surfaces. Ni-MOF@NF materials have found broad applications, including fuel cells [44]. Ultrasonication and solvothermal/hydrothermal methods can be combined to grow MOFs on highly porous carbon materials for microwave absorption applications [45]. Ligands/MOF linkers are not necessarily limited to a single compound. In an elegant contribution [46], multivariate film MOFs were formed on the surface of conductive fluorine-doped tin oxide (FTO) by reacting metal ions with different ratios of pyromellitic diimide bis-pyrazolate (PMDI) and naphthalene diimide bis-pyrazolate (NDI) in the presence of cleaned FTO substrates. The resultant MOFs answered some fundamental questions about diffusional electron hopping transport and redox conductivity. Here, coating MOFs on the FTO is critical for electrochemistry studies. The Ni-MOF coating on a stainless steel substrate has also been demonstrated using a simple solvothermal method [47], where Ni^2+^ and ligand were mixed in an *N*,*N*- dimethylformamide and ethanol solvent mixture (6:2) in a glass vial, followed by inserting a clean stainless steel piece and heating the mixture at 90 °C for 24 h. The resultant binder-free Ni-MOF thin film turns out to be a supercapacitor electrode. Another example of coating MOFs on stainless steel surfaces also utilized solvothermal synthesis to grow Ni/Co-based nanosheet-like MOFs on a clean stainless-steel substrate for use as a high-performance supercapacitor [48]. Copper substrates can also be coated with MOFs, as demonstrated by a porphyrinic MOF thin film growth, where atomic layer deposition (ALD) and pseudomorphic replication (PMR) were combined to ensure the formation of a thin film [49]. A universal strategy that can be applied to different substrates was also reported in ref. [50], which allows for the in situ growth of large-area and continuous MOF films with controllable microstructures. The key innovation is to modify the surfaces of various substrates, including glass, stainless steel wires, capillaries, and MOFs, with poly(4-vinylpyridine) as the anchor to capture metal ions via Coulomb attraction. This strategy will significantly broaden the use of MOFs as coatings on desired substrates to achieve the needed functions.

Drawbacks of the in situ (solvothermal/hydrothermal) growth of MOFs on substrates are also recognized. For example, the request for high temperature and pressure (in a sealed container) limits the coating of temperature-sensitive substrates with MOFs. It may also be difficult to control the film thickness and crystal morphology, resulting in non-uniform coatings. Reactions are also slow, limiting industrial-scale production or high-throughput fabrication. Many substrates (especially glass and polymers) require surface pretreatment to initiate nucleation, increasing the cost and complexity. This method also often involves toxic or high-boiling-point solvents (e.g., DMF) in large volumes, raising environmental concerns and complicating post-synthesis disposal. Uneven ligand diffusion and nucleation may also result in poor uniformity, limiting advanced microstructure or device design. It may also be difficult to control crystal orientations. Nevertheless, the in situ (solvothermal/hydrothermal) growth of MOFs is still widely applied whenever possible due to its straightforward principles and operations.

### 2.3. Layer-by-Layer Deposition

**Figure 4:**
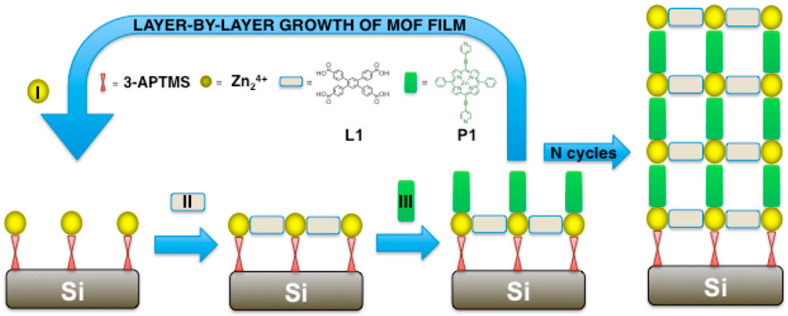
Representative scheme of the Layer-by-Layer (LbL) growth of the DA-MOF and L2-MOF structures on idealized 3-APTMS, resulting in a film after N cycles of growth. Reproduced with permission from Ref. [52]. Copyright 2013, American Chemical Society.

Layer-by-layer (LbL) assembly is a particularly useful coating approach, often referred to as atomic layer deposition (ALD) and molecular layer deposition (MLD). LbL assembly is a highly controlled, sequential deposition of thin film technique that builds up materials one atomic or molecular layer at a time (Figure 4) [51]. The key to LbL assembly is to expose the substrate surface to alternative precursors or materials so that each can only react with the present layer, offering only a monolayer deposition per cycle. Inert gas purge is usually required between layers. ALD allows for 0.1–0.3 nm of materials to be coated each cycle, while MLD involves organic precursors to create polymeric or hybrid chains. LbL assembly offers a unique advantage of atomic-level control of film thickness with exceptional uniformity even on complex geometries. MLD offers enhanced tunability in composition and coating functionality, and is scalable and reproducible for both laboratory and industrial applications. Traditionally, ALD is used in advanced microelectronics for precise, uniform coatings, while MLD is used in hybrid organic–inorganic functional films for energy and materials applications. LbL assembly has recently been adapted to surface-mounted MOFs.

MLD is particularly useful for LbL deposition for MOF synthesis because the metal ions and ligand molecules can be alternatively applied to the substrate surface, forming highly uniform MOFs with controlled thickness and orientation, as well as reduced thin film defects and film roughness regardless of surface geometry. Depending on how metal ions and linkers/ligands are applied, LbL assembly can be achieved by several methods such as dipping, pumping, spraying, spin-coating, vacuum filtration, solution ALD, and autoclave synthesis [20,52,53,54,55,56,57,58]. Regardless of the LbL method of chosen, it is critical to include a cleaning or rinsing process between applying two adjacent layers, in order to avoid cross contamination of the linker or metal ions and to prevent formation of MOF nanocrystals on substrate surface composed of residual linkers or metal ions on the substrate or inside the pores of the existing MOF layer. Substrates are also often required to undergo certain pretreatments via at least rinsing with organic solvents and drying with nitrogen; other functional groups may need to be preanchored to ensure the coating of the first layer. A typical cycle usually contains alternating application of metal ion solution, pure ethanol, ligand solution, and pure ethanol again. Depending on the time allowed and the particular needs, a few cycles to tens of cycles have been reported. Being able to precisely control each layer also makes it possible to mix the metal ions or linkers among different layers to obtain advanced optical or electrochemical properties through the formation of heteroepitaxial interfaces [59].

The MOF structures formed by LbL assembly also depend on the feeding ratio of metal-to-linker. For example, in ref. [60], different Zn-BPDC MOF structures were formed on a pretreated silicon substrate at 55 °C with varied metal-to-linker ratios through a spray LbL approach. The impact of the number of cycles was investigated on a Cu-BDC MOF deposited on an ethanol-rinsed silicon substrate using LbL assembly with a metal (Cu(CO_2_CH_3_)_2_·H_2_O) and a linker precursor solution (1,4-benzenedicarboxylicacid (H_2_bdc) and 1,4-diazabicyclo [2.2.2]octane (DABCO) in ethanol) at 62 °C with a rinsing procedure in between [61]. Both the orientation and phase are affected by the number of cycles. In another contribution [62], an LbL chemical vapor deposition (CVD) was applied to form ultra-smooth conductive MOF thin films on a liquid gallium substrate. The preparation of a heterostructured thin MOF film was demonstrated in a recent contribution [63], wherein a pretreated and functionalized FTO substrate was immersed in Cu(OAc)_2_ in DMF at 57 °C for 30 min, followed by washing with DMF, immersion in BPDC linker at 57 °C for 30 min, ending with wash with DMF and drying by N_2_ gas. Forty cycles were found to form a uniform Cu-BPyDC thin film (~400 nm). Then, this MOF was dipped into ethanolic solutions of Cu(OAc)_2_.H_2_O and TCNQ for 5 min each under elevated temperature for 5 cycles. A charge transfer between the two insulating MOFs was demonstrated with mechanisms revealed based on first principles theory. In another elegant contribution [64], automated LbL deposition was demonstrated on perylene diimide linkers and Cu^2+^ to synthesize MOFs as oriented films with uniform and predefined thickness on glass and silicon platforms. The key to the automation relies on a computer-controlled automated pump system that exposes the pretreated substrate to each precursor solution at 50 °C. The substrates were successively exposed to 100 μM zinc(II) acetate (or copper(II) acetate) solution, 2 μM PDI(COOH)2, and 20 μM BIPY or DABCO ethanolic solutions, each for 15 min. Between each exposure, the substrates were soaked twice with absolute ethanol for 7 min each to remove physisorbed reactants. The accuracy of the thickness of LbL deposition for MOF thin film synthesis can be as low as a single molecular level, as demonstrated in a recent procedure wherein a Cu-BDC MOF thin film was prepared [65]. The key to achieving such a high accuracy is solution ALD (sALD), whose growth rate was found to be 4.5 Å per cycle. In comparison to dipping or spray LbL methods, sALD offers a superior film thickness control and uniformity. This work also proves the highly controlled LbL MOF growth mechanism. To date, a number of MOF thin films have been synthesized by various LbL methods using different solvents; a comprehensive table is available in a recent review article [20].

Although LbL deposition can offer superior accuracy in thin film coating growth, precise thickness control, and high uniformity, it can be time-consuming when thick films are needed. ALD or MLD requires vacuum equipment and volatile precursors, which may not be easily accessible to common research labs. The formed MOF films may be limited by the availability of suitable precursors and the stability of the film during deposition. Nevertheless, LbL deposition is a unique and powerful tool for synthesizing the desired MOF thin films.

### 2.4. Vapor Phase Deposition

Vapor phase deposition is another commonly used method to deposit thin films or coatings onto a substrate surface. As indicated by its name, this method relies on vaporized chemicals or converting the target materials into vapor and carrying out the deposition reaction on the substrate surface before condensation. Two vapor phase deposition methods are often seen, namely physical vapor deposition (PVD) and chemical vapor deposition (CVD). Vapor phase deposition is commonly used in semiconductor fabrication, optical coatings, decorative coatings, and solar cell manufacturing. Recently, vapor phase deposition, especially CVD, has been applied to MOF thin film production, leveraging precise control over film thickness and composition, high purity, uniform coating, and endorsing electrical or optical properties to the coatings.

To carry out vapor phase methods for MOF thin film synthesis, it is critical to evaporate or sublimate MOF precursors in a controlled manner, followed by their reaction and condensation on a substrate surface. To date, four vapor phase MOF preparation methods have been developed, including vapor-assisted conversion (VAC), vapor-phase transformation (VPT) of metal oxide films to MOF films, vapor-phase linker exchange (VPLE), and atomic layer deposition (ALD)/molecular layer deposition (MLD), which offer good crystallinity, shape retention, multifunctionality, and thickness controllability, respectively.

**Figure 5:**
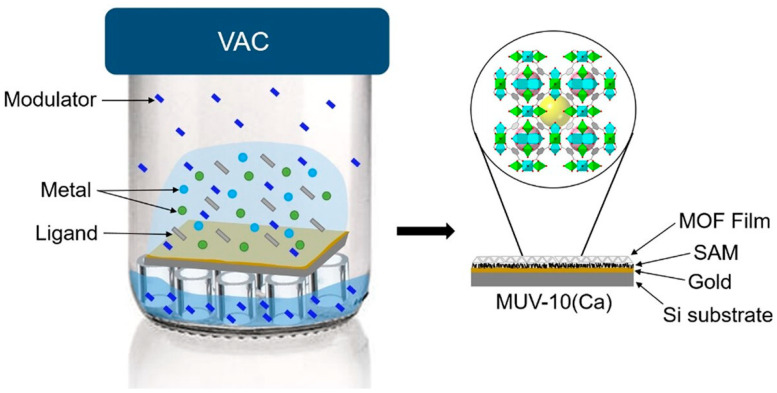
Representative scheme of the VAC method for MUV-10(Ca) thin film fabrication. Reproduced with permission from Ref. [66]. Licensed under CC-BY 4.0 with no change made. https://creativecommons.org/licenses/by/4.0/ (accessed on 1 May 2025).

VAC relies on heating the precursors from solution to a moderate temperature to generate vapor with the desired composition (Figure 5) [66]. Such evaporated precursors then interact with the solvent vapor to crystallize into a continuous, crystalline, and porous thin film. Starting with the first VAC-synthesized MOF, ZIF-8, and ZIF-67, where crystallization of the precursors occurred after H_2_O vaporization at a high temperature, a number of MOF thin films have been synthesized in this way, including PCN-6, UiO series, CAT-1, Co-MOF-74, Ni-MOF-74, Mg-MOF-74, and PCN-222 films [67]. The advantages of VAC include the preparation of highly crystalline MOF films (via controlling the precursors and solvent ratio on a large scale), mild reaction conditions in comparison to the classic solvothermal phase method, and minimized solvent molecules on the MOF film. However, VAC requires a solvent and is challenged by corrosion and chemical contamination. Also, nonuniform crystallization and reactivity variations in the precursors can limit the control of the synthesized MOF films.

VPT does not involve the use of solvents or induce corrosion because the organic linker of the target MOF can be supplied as a vapor instead of as a gas evaporated from a solution. The properties of the formed MOF films, such as morphology, thickness, and location, depend on the metal oxide film precursor, which has to match the oxide dissolution and MOF crystallization rates. Usually, CVD or PVD methods are applied to create a thin layer of metal oxides, followed by transforming the metal oxide films into MOF films through vapor phase conversion technology, offering superior shape retention and controllability. ZIF-8, ZIF-61, ZIF-67, ZIF-72, Cu-CDC, Cu-BDC, and HKUST-1 thin films have been synthesized using VPT. Concerns related to VPT-based MOF synthesis lie in substantial deformation with thickness increase during the reaction, resulting in internal stress on the film and reduced mechanical stability. VPT also requires relatively demanding preparation conditions.

VPLE relies on reacting to an organic linker vapor with an existing inorganic–organic hybrid or MOF films and offers minimal film deformation during the conversion of linkers due to the minimal change in the thickness of the film after the exchange. ZIF-8, ZIF-8/I, ZIF-8/Br, and carboxylate MOFs are typical examples of MOF thin films obtained via VPLE. VPLE offers the possibility to modify MOF thin film properties allowing for the preparation of multifunctional MOFs by adjusting the mixing ratio. Further investigation may be needed to reveal the mechanism governing the hybridization degree in the resultant MOF.

**Figure 6:**
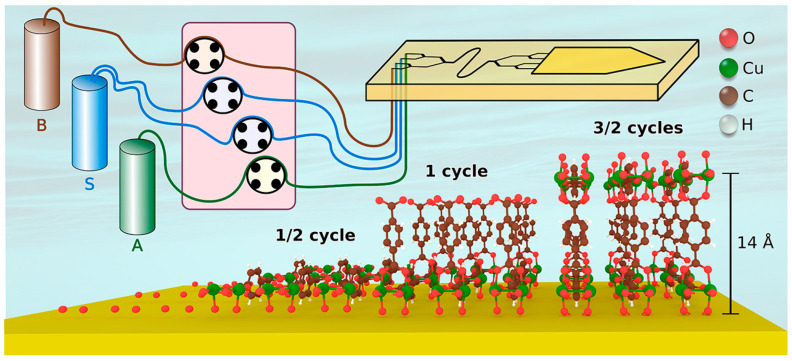
Representative scheme of the sALD setup outlining the peristaltic pump arrangement with the connections to the reaction chamber and the growth of Cu-BDC. A corresponds to the first precursor path (Cu_2_(OAc)_4_), B to the second precursor path (terephthalic acid), and S to the solvent path (ethanol). Furthermore, the size of the Cu-BDC unit cell of 14 Å is indicated. Reproduced with permission from Ref. [65]. Licensed under CC-BY-NC-ND 4.0 with no changes made. https://creativecommons.org/licenses/by-nc-nd/4.0/ (accessed on 3 May 2025).

ALD/MLD is essentially LbL assembly in the vapor phase, and thus offers all the advantages that LbL methods can offer. A number of MOFs have been prepared in this way, such as MOF-5 nanofilms, NU-1000, UiO-66, ZIF-8, Cu-TPA, and manganese- and cobalt-based MOF films. In vapor-phase ALD/MLD, film thickness is controlled by tuning the precursors, temperature, and the number of cycles. The challenges of vapor phase ALD/MLD lie in improving crystallinity and broadening the range of feasible MOF films (Figure 6) [65].

Although quite innovative and promising, vapor phase deposition faces certain challenges. For example, it requires volatile and thermally stable precursors, a condition that may not be met by every MOF that is needed. It may also be challenging to fine-tune the stoichiometry and crystallinity of the formed MOF thin films. Furthermore, regardless of the vapor phase deposition method of choice, specialized and often expensive equipment is needed. Nevertheless, vapor phase deposition is a powerful method to synthesize MOF films with high purity and density as well as high scalability, if the precursor of the target MOF is thermally stable.

### 2.5. Other Coating Methods

Previous coating methods rely on the coordination reaction between metal ions and ligands at the surface of interest with the assistance of seed layers, binders, or templates. It is also possible to directly synthesize MOFs on substrate surfaces (without seed layers) or directly deposit preformed MOFs onto substrate surfaces. In the latter case, presynthesized MOFs are directly applied as a slurry or paste onto the target substrate. The advantages of such direct coating include a simpler process, fewer number of steps (and thus less complexity), and, in most cases, strong adhesion of the resultant coatings. Substrate compatibility, on the other hand, needs extra caution, together with good control over the nucleation process (if uniform coatings are needed). Here we aim to summarize a few methods that are commonly seen in MOF-based coating research due to space limitation.

#### 2.5.1. Spin-Coating

**Figure 7:**
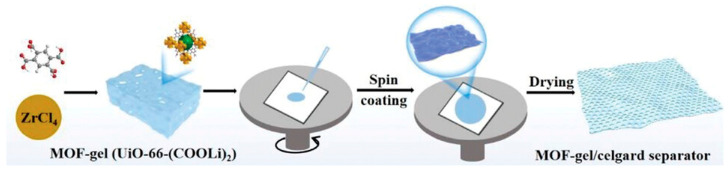
Representative scheme of the synthesis of MOF-gel and the subsequent film fabrication process through the spin-coating method. Reproduced with permission from Ref. [68]. Copyright 2024, Wiley-VCH GmbH.

Spin-coating, as is evident by the name, relies on spreading MOF solutions or slurries over rotating substrate surfaces via centrifugal force (while allowing the solvent to evaporate), resulting in a thin film of an MOF (Figure 7) [68]. Typical spin-coating involves the following four key steps: (i) Dispensing: a small volume of coating liquids (solution, sol–gel, polymer, etc.) is dropped onto the center of the substrate; (ii) Spreading: the substrate is rapidly rotated, typically at 1000–10,000 RPM to spread the liquid via centrifugal force; (iii) Thinning: excess fluid is pushed toward the edges by the centrifugal force while the remaining liquid becomes thinner uniformly; (iv) Evaporation: the solvent evaporates during spinning, leaving behind a solid film. The thickness of the resultant film depends on (a) the spin speed (the higher the RPM, the thinner the film), (b) the viscosity of the solution (the higher the viscosity, the thicker the film), (c) the spin time (the longer the spin time, the thinner the film), and (d) the solvent volatility (the more volatile the solvent, the thinner the film). Spin-coating is best for uniform films on flat, circular substrates, and is traditionally adapted in OLEDs and displays, in anti-reflective coatings for lenses and optical devices, in quantum dots, and in polymer films as sensors and membranes.

For adaptation to MOFs and relevant composites, uniform, nano-sized MOFs (<200 nm) are ideal for smooth films while lower concentrations yield smoother, thinner films. Substrates may require pretreatment or seed layers to promote MOF attachment. If thicker films are needed, multiple spin-coat cycles may be necessary. It should be noted that spin-coating is often combined with other approaches. For example, a recent work demonstrated the formation of highly oriented MOF thin films by combining spin-coating and liquid-phase epitaxy (LPE) in a high-throughput fashion [55]. Various MOF thin films with a thickness of a few micrometers to nanometer scale have been demonstrated on benchmark MOFs, such as Cu_2_(bdc)_2_•xH_2_O, Zn_2_(bdc)_2_•xH_2_O, HKUST1, and ZIF8. The method has been proved effective on a variety of substrates, including gold, silicon, glass, porous stainless steel, and aluminum oxide. Each surface was preprocessed differently to ensure adhesion, followed by spin-coating of metal ions for 5 s and ligand for 8–10 s. All substrates are placed on a rotating stage. The advantages include shortened preparation time, lower chemical and solvent consumption, and cost-effectiveness in comparison to traditional LPE. Other recent spin-coating works include the formation of CoCe-BTC/PEDOT composite films on an interdigital electrode (IDE), HKUST-1 on Menzel glass, and ZIF-8 and MIL-53(Al) on a conductive MOF surface [69,70,71]. These applications demonstrate the use of spin-coating in forming thin films on various surfaces. An interesting finding, although it does not involve MOFs being coated, it is worth mentioning that, in combination with polymer-assisted deposition (PAD), spin-coating was applied to generate a thin film MoS_2_ with precise thickness control on a SiO_2_/Si wafer. The key to ensuring adhesion was the use of a complex polymer-assisted precursor, a precursor−polymer complex thin film comprising anhydrous ammonium tetrathiomolybdate (ATM) precursor and linear-poly(ethylenimine) (L-PEI) [72].

Spin-coating is relatively simple to operate, rapid (coating and drying can be completed in seconds), scalable, and cost-effective. It also often results in more uniform thin films, especially on flat substrates. It offers precise thickness control (sub-micrometer to nm range) and requires a minimal amount of solution. The reproducibility is also high if coating conditions are well-controlled. Cautions when conducting spin-coating include target surface size (to ensure relative spinning between MOFs and substrate) and curvature (flat surfaces are preferred). The viscosity of the MOF slurry or solution, as well as the spinning rate need to be considered when highly controlled coating products are needed. It can be challenging for irregular substrate shapes and may result in thicker rims. The solution that is spun off can be wasted if no proper ways are available for recycling and reuse. If the solvents are highly volatile, coating defects can occur.

#### 2.5.2. Dip-Coating

**Figure 8:**
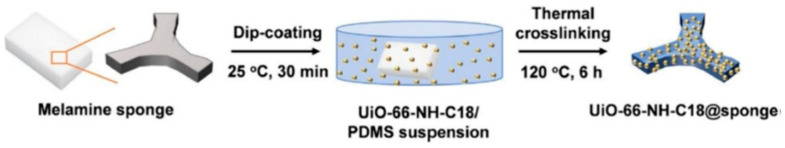
Representative scheme of the preparation of the MOF UiO-66-NH-C18@sponge by the dip-coating method. Reproduced with permission from Ref. [73]. Licensed under CC-BY 4.0 with no change made. https://creativecommons.org/licenses/by/4.0/ (accessed on 5 May 2025).

Dip-coating is another way of directly coating presynthesized MOFs onto target substrates (Figure 8) [73]. Traditionally, dip-coating materials are commonly seen as solutions, suspensions, or colloids. As a target substrate is dipped into these materials and removed at a controlled and often optimized rate, a thin layer of coating materials is formed as the solvents evaporate. Dip-coating consists of the following five sequential steps: (i) Immersion: the substrate is dipped into a solution containing the coating material; (ii) Dwell Time: the substrate remains in the solution for a specific time duration to allow for adsorption and wetting; (iii) Withdrawal: the substrate is then pulled out at a controlled rate, which directly influences the final film thickness; (iv) Drainage: excess liquid needs to be drained from the substrate under proper (and controlled) gravity and viscosity; and (v) Drying/Solidification: the solvents are evaporated to let the coating cure and leave a uniform film. According to the Landau–Levich–Derjaguin theory, the coating thickness depends on withdrawal speed (V), fluid viscosity (η), surface tension (γ), density (ρ), and gravitational and capillary forces.

Classic dip-coating materials include polymers, sol–gel precursors, metal oxides, nanomaterials, biomaterials, and hydrogels. Recently, preformed MOFs have also been coated onto various substrates using dip-coating. Because MOFs can sediment quickly, keeping MOF particles suspended is critical for dip-coating, which may be achieved by using surfactants. Poor MOF adhesion is another concern, which may be overcome by prefunctionalization of the substrates. To fix MOF crystals and to improve crystallinity, mild heating or solvothermal crystallization may be required. A typical example is coating UiO-67 onto Ce(OH)_4_-PIM polymer sheets by preparing a UiO-67 suspension in methanol and dipping the Ce(OH)_4_-PIM in for 10 min (one cycle) [74]. The resulting composites were then dried overnight. Varied dipping times and cycles were tested. The quality of the resulting film on the polymer sheets was not as good as the UiO-67 film coated on the same substrate through other coating methods (such as drop-casting—see below). This outcome may be caused by the low mechanical stability of the polymer sheets, the non-uniform UiO-67 suspension when a longer coating time was employed, and non-uniform contact between the sheets with the UiO-67 suspension. On a mechanically stable substrate, such as an FTO glass, dip-coating showed much greater performance. For example, as demonstrated in ref. [75], an etched FTO glass substrate was dipped into a Cu(Oac)_2_ precursor in EtOH for 20 s, washed for 5 s with EtOH to remove unreacted products, immersed in HHTP precursor in EtOH for 40 s, and washed again with EtOH for 5 s. This cycle was repeated 50 times until a unique, thick film was coated on the FTO glass.

The advantages of dip-coating are the simple operation, scalability, and applicability to substrates with complex shapes. Minimal equipment is needed. Dip-coating also offers the opportunity to produce smooth and consistent films with high reproducibility. Substrates with complex geometries can often be coated via dip-coating. However, the coating/film quality can be inconsistent, as gravitational effects can affect the coating process. When drying is needed to remove solvents, the thermal stability of the substrates also needs to be considered. Dip-coating can be challenging when very thick films are needed. Drying and curing times can be long, during which dust, vibration, or non-uniform withdrawal can cause uneven coating surfaces. Environmental concerns about the use of volatile solvents may require fume hoods or similar setups.

#### 2.5.3. Spray-Coating

**Figure 9:**
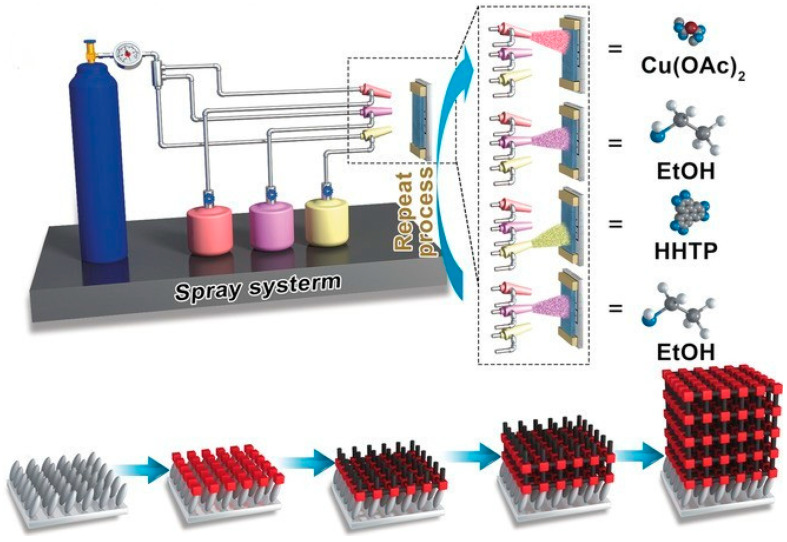
Representative scheme of the preparation of MOF Cu_3_(HHTP)_2_ thin-film gas sensors by the spray-coating strategy. Reproduced with permission from Ref. [76]. Copyright 2017, Wiley-VCH Verlag GmbH & Co. KGaA, Weinheim, Germany.

Spray-coating is also a commonly used direct coating approach. It involves spraying, with compressed air or other thermal/plasma approaches, fine droplets of solutions, suspensions, or melted materials onto the target surface to form a thin, ideally uniform film after drying or curing (Figure 9) [76]. It often atomizes a liquid (solution, suspension, or melt) into a mist or aerosol and transports it onto a substrate using pressurized gas, air, or electrical/magnetic fields. The droplets deposit and spread on the surface, followed by drying or curing to form a continuous film. The following four steps are important for spray-coating: (i) Atomization: breaking the liquid into small droplets using a nozzle; (ii) Transport/spray: moving the droplets to the target surface via air or other forces; (iii) Deposition: droplets hit the surface and spread; and (iv) Drying/Curing: solvents are evaporated to form a film. Depending on the atomization mechanism, several types of spray-coating are available, such as air spray (common/conventional), airless spray (high fluid pressure), electrospray (using electric fields to create aerosols), ultrasonic spray (high-frequency vibration), and thermal spray. Key parameters for spray-coating include (a) spray nozzle design (critical for droplet size and pattern), (b) atomization pressure (important for droplet velocity and coating coverage), (c) solution viscosity, (d) substrate distance (essential for droplet drying and film smoothness), and (e) substrate temperature (critical for adhesion and evaporation).

Classic spray-coating materials include polymers, sol–gel precursors, metal oxides, nanomaterials, biomaterials, paints, inks, dyes, and hydrogels. Recently, MOFs have also been coated on substrates using spray-coating. When MOFs need to be coated on a particular surface, the solvent drying rate needs to be considered, as fast drying usually prevents MOF particle adherence while slow drying can result in coalescence and dripping. Pretreatment may help anchor MOFs. For heat-sensitive MOFs, hot spraying should be avoided. For example, as demonstrated in ref. [77], UiO-66 nanoparticles (50 to 500 mg) were added to 1 g of waterborne polyurethane (WPU) in water under stirring to make a uniform suspension. Glass slides, the substrates involved in this study, were sonicated in isopropanol and water for 15 min each, followed by drying with an N_2_ flow. The UiO-66/WPU mixture was then sprayed on the glass slides at 2.5 bar from 15 cm away using an air gun. The glass was fixed vertically on a hot plate at 90 °C to evaporate the water. The coated glass was then cured at 90 °C for 1 h. Lastly, the surface was modified by spraying a 1% solution of a 70:30 isopropanol/water mixture followed by curing at 120 °C for 2 h. This procedure also works for other surface substrates, such as metals and plastics. The intercalation of polyurethane chains immersed in MOF pores results in excellent robustness of the coating films. Spray-coating can also be combined with LbL and LPE deposition to enhance the uniformity of the resultant films, wherein the metal ion and ligand precursors are sprayed on the target surface alternatively. For example, as demonstrated in ref. [78], a Zn-TPE thin film was formed via LPE LbL spray-coating on a quartz substrate. The substrate was maintained under 55 °C. Then, a 1 mM Zn (NO_3_)_2_ DMF solution was sprayed for 25 s, followed by a 10 s pause, and washed for 5 s. The 0.1 mM TPE DMF solution was then sprayed for 35 s, another pause for 10 s, and another wash for 5 s. This completes 1 cycle, and a total of 20 cycles were carried out. The resultant composite was then immersed in EtOH for 1 min and dried with N_2_. Similarly, in ref. [79], Cu_3_(HHTP)_2_ was spray-coated with LPE LbL on sapphire and Si/SiO_2_ substrates with different spray, pause, and drying times, as well as precursor concentrations. Both works resulted in highly uniform films with desired functions (see Section 3: Representative Applications of MOF Coatings).

The advantages of spray-coating include the rapid operation and suitability for large and complex surfaces (such as flat, curved, and/or irregular surfaces). The thickness can be tuned by time and passes/rounds of spray. The lack of contact with the substrates makes spray-coating ideal for delicate substrates. The limitations include the uniformity of the films, which depends heavily on spray technique, and potential material waste. Fume control can be critical for volatile solvents. Drying of the droplets may lead to defects, while the process can be sensitive to humidity, temperature, and droplet size.

#### 2.5.4. Drop-Casting

**Figure 10:**
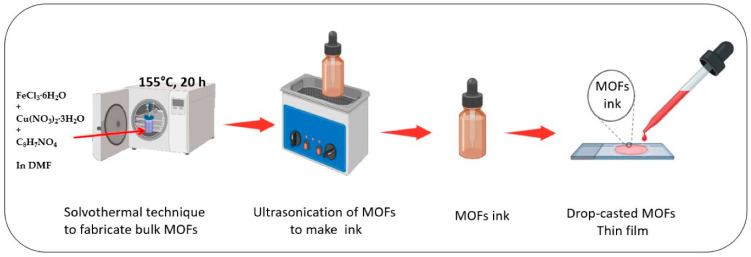
Representative scheme of the MOF thin film fabrication via the drop-cast technique. Reproduced with permission from Ref. [80]. Copyright 2023, MDPI, open access.

Drop-casting has also been applied in coating. It involves placing a droplet (or multiple droplets) of liquid solution or suspension on the target surface, followed by drying the surface naturally or under controlled conditions (Figure 10) [80]. It is commonly used to coat polymers, nanomaterials, metal oxides, and biomaterials onto common substrates, such as glass slides, plastic films, and metal foils. Recently, it has been applied to coat MOFs onto solid surfaces. The advantages include ease of operation and the ability to coat complex materials on target. Limitations are also present, such as the difficulty in controlling thickness and uniformity, cracking in the resultant films, and small-scale operations. For example, as demonstrated in ref. [74], a UiO-67 solution was suspended in 90% EtOH/10% Chloroform, followed by casting the UiO-67 droplets onto Ce(OH)_4_-PIM polymer sheets, whose surface was slightly wet, to avoid any initial electrostatic repulsion and to smooth the surface to enhance the uniformity of the resultant thin film, and more uniform compared to the MOF produced via dip-coating. Another notable point of this work is that it compared different direct coating methods on the same polymer sheets to identify the best coating strategy.

### 2.6. MOF/Substrate Interface Engineering

The interface between an MOF and its target substrate is critical for the overall performance, stability, and functionality of the coating. While weak interfacial adhesion can result in detachment of the coating layers, a poorly engineered interface may hinder efficient charge or mass transport, thereby reducing the performance of MOF-based devices. Thus, precise interface engineering is essential to ensure the reliable incorporation/integration of MOFs into the functional systems of the target surfaces. To this end, most target surfaces have to undergo pretreatment before applying MOF coatings through surface functionalization and modification strategies aimed at enhancing MOF nucleation and adhesion. Chemical treatments with acids, bases, or reactive reagents can alter the substrate’s surface energy, smoothness/roughness, and create active sites for MOF growth. Plasma treatments, on the other hand, modify both the surface topography and chemistry, improving wettability and enabling the introduction of functional groups. Silanization and grafting, where functional organic molecules, such as silanes, are covalently bound to the substrate surface, provide the desired/controlled interaction sites for the MOF precursors, promoting controlled orientation and strong adhesion.

To better prepare the substrate surface for coating, understanding interfacial interaction/bonding mechanisms becomes essential for MOF coating. The commonly seen interactions may span from weak physical adsorption (e.g., van der Waals forces), to electrostatic attractions between oppositely charged components, to stronger chemical bonding involving covalent or ionic linkages, and even to mechanical interlocking, wherein MOF nanoparticles are physically anchored within the infrastructures of substrate surfaces. To further enhance the adhesion, uniformity, and durability of the MOF coating, various strategies can be employed, including but not limited to the deposition of seed layers (e.g., metal oxides) to facilitate nucleation, the use of adhesion promoters that interact with both the substrate and the MOF, and the design of hierarchical surface structures to improve mechanical interlocking and to increase the effective surface area. Post-deposition treatments, such as annealing or chemical modification, can also reinforce interfacial stability.

A comprehensive understanding of the MOF/substrate interface requires advanced characterization techniques. Scanning electron microscopy (SEM) and transmission electron microscopy (TEM) provide insights into surface morphology, coating thickness, and crystallographic details. Atomic force microscopy (AFM) enables the evaluation of surface roughness, adhesion forces, and mechanical behavior at the nanoscale. X-ray photoelectron spectroscopy (XPS) reveals elemental composition and chemical states, while Fourier transform infrared spectroscopy (FTIR) identifies interfacial functional groups and bonding mechanisms. Together, these tools help elucidate the complex interactions at the MOF/substrate interface and guide the rational design of robust, high-performance MOF coatings.

## 3. Representative Applications of MOF Coatings

Coating MOFs on electrodes, polymer films, and glass surfaces has found broad applications in energy storage, supercapacitors, electrochemical sensors, biosensors, gas sensors, corrosion resistance, and biomedical applications. Several examples of the application of MOF coatings have been discussed in Section 2. Here we will highlight a few more examples focusing on the areas listed above. Our goal is not to cover every published paper on the applications of MOF coatings. Instead, we aim to show the cases of typical applications in different areas, with details on how MOFs are coated onto the corresponding surfaces, why a coating method is selected, what specialty MOF coatings offer, and what the major achievements are in each example using MOF coatings in the past 3–5 years. More extensive applications can be found in the relevant reviews.

### 3.1. Batteries

**Figure 11:**
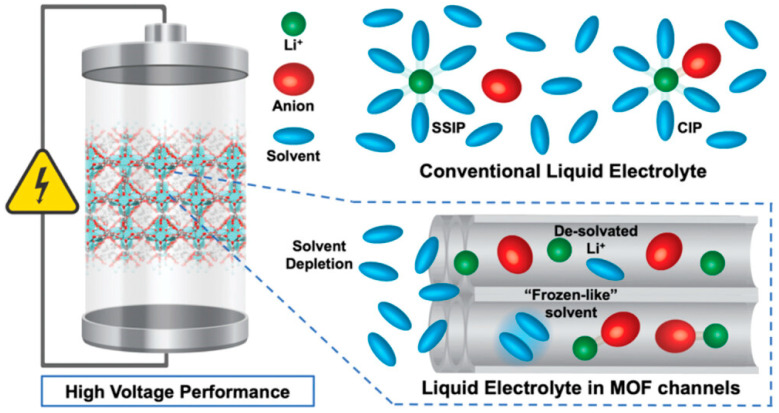
Representative scheme showing the MOF-based electrolytes used to increase the operating voltage range of batteries. Reproduced with permission from Ref. [81]. Open access. Licensed under CC BY 4.0 Attribution 4.0 International Deed, with no changes made. https://doi.org/10.1002/advs.202305280.

MOF-based nanocomposites have been adapted to promote battery technology (Figure 11) [81]. In a recent elegant contribution [32], Co-MOF was coated onto a conductive substrate, Ni foam, to fabricate a binder-free electrode. The coating method was cathodic electrodeposition, wherein Co^2+^ ions and 1,2 benzene dicarboxylic acid (BDC) were mixed in a DMF/water mixture, followed by electrodeposition using the chronoamperometry technique. At the cathode, NO_3_^−^ is reduced and OH^−^ is generated, which then deprotonates BDC to BDC^−^, ready for forming MOFs with Co^2+^ on the conductive substrate. Here, the electrodeposition method is ideal for the formation of a thin layer of conductive films. The resultant Co-MOF coating offers the synergistic effects (metal ions and ligands), which resulted in high electrolytic diffusion and charge transfer at the interface of electrode and electrolyte. An energy density of 63.06 Whkg^−1^ at a power density of 479.94 W kg^−1^ was achieved, which suppresses most previously reported MOF-based devices. It also shows a capacitance retention of 99.6% over 50,000 cycles.

In another contribution [44], Ni-MOF was synthesized and deposited on Ni foam and used as a bifunctional electrocatalyst for direct urea and nitrate fuel cells. The Ni-MOF was first synthesized via the solvothermal method, followed by a dip-coating method to fabricate Ni-MOFs on a nickel foam (NF) electrode. The NF was ultrasonically cleaned in ethanol and DI water to remove surface contaminants and impurities. The prepared Ni-MOF was then dispersed into ethanol via ultrasonication, followed by dip-coating onto the dried, cleaned NF, which was dried for 10 h at 100 °C to obtain the Ni-MOF electrode. The resultant coated electrode showed a peak current density of 188 mA/cm^2^ for urea oxidation reaction (UOR) and −14 mA/cm^2^ for nitrate reduction reaction (NRR) at an onset potential of ∼1.58 V (vs. RHE), and ∼1.12 V (vs. RHE), respectively, due to the multiple oxidation states of Ni (i.e., Ni^2+/3+^) and high conductivity of the organic ligands. The electrodes retained ∼71.2% and ∼83.9% capacity after 20,000 s of UOR and NRR, respectively.

MOFs can also be used as nanofillers to improve the performance of batteries. For example, in ref. [82], a Co-based MOF, ZIF-67, was incorporated into a PEO matrix and interacted with lithium bis(trifluoromethanesulfonyl)imide (LiTFSI). A high initial capacity of 130 mAh g^−1^ and capacity retention of 85 mAh g^−1^ after 80 cycles were achieved at 0.2 °C and 60 °C.

### 3.2. Supercapacitor

**Figure 12:**
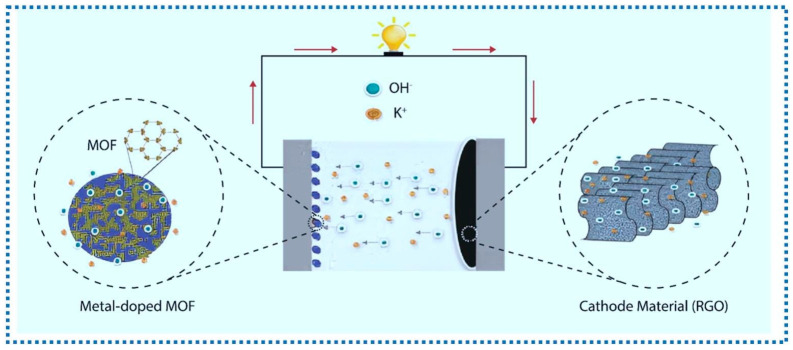
Representative scheme of the operational mechanism of supercapacitors based on metal–organic framework (MOF) materials. Reproduced with permission from Ref. [83]. Licensed under CC BY-NC-ND 4.0 Attribution-Non Commercial-No Derivatives 4.0 International Deed with no changed made. https://doi.org/10.1016/j.est.2023.109822.

The high porosity and abundant redox capacitive sites for charge movement of MOFs make them great candidates as electrode materials in supercapacitors. MOFs can also be doped to introduce defects and to alter the electronic structure of the host MOFs (Figure 12) [83], leading to advanced surface reactivity and electrochemical properties. An elegant example is a Cu doped Fe-MOF coated as a thin film on Ni foam via a simple drop-cast method [80]. Upon preparation of the powder of Cu@Fe-MOF electrode in DMF by the solvothermal method, this MOF powder was dispersed in anhydrous DMF and ultrasonicated for 2 h. The resultant, homogenous ink was then drop-cast onto a conducting NF substrate. Finally, the drop-cast coated Cu@Fe-MOF/NF film and Fe-MOF/NF electrodes were dried at 50 °C immediately to produce an active surface. Here, the drop-casting method is selected due to its wide adaptability in device preparation. The resulting device demonstrated a high specific capacitance of 420.54 F g^−1^ at 3 A g^−1^, twice that of the nano-cuboidal Fe-MOF/NF (210 F g^−1^). The energy density, 44.20 Wh kg^−1^, is also excellent, with an 88% capacitance retention after 5000 cycles.

In another example, Ni-MOF was coated onto Ni_3_S_2_/Ni foam surfaces, providing a large specific area and porosity [42]. In brief, upon a successive cleaning of NF with acetone, HCl aqueous solution, deionized water, and ethanol under sonication and drying in a vacuum oven, Ni_3_S_2_ was synthesized on the resultant NF. Then, the NF with in situ synthesized Ni_3_S_2_ and a Ni(NO_3_)_2_·6H_2_O and PTA mixture was heated, resulting in a deposition of Ni-MOF onto Ni_3_S_2_. Here, the solvothermal in situ growth method allows for the complex substrate (NF with in situ synthesized Ni_3_S_2_) to be coated. An outstanding specific capacitance, 2207 F g^−1^ at 1 A g^−1^, 1929.7 F g^−1^ at 5 mV s^−1^, was obtained and retained, 62.3% at 10 A g^−1^ as well as 90.4% at 50 mV s^−1^, after 5000 cycles. The device also displayed a high energy density at 24.5 Wh kg^−1^ at a power density of 375 W kg^−1^. A relevant contribution examines the effect of solvothermal temperature on the supercapacitor performance of Ni-MOF on Ni foam, and found that 80 °C offered a larger specific surface area with a cross-network structure formed on its surface, likely the reason to result in a specific capacity of 30.89 mA h g^−1^ under a current density of 1 A g^−1^ [43].

A conductive 2D NiPc-MOF was uniformly coated onto Ni foam through in situ anodic electrodeposition (AED) as a flexible supercapacitor [84]. The in situ growth AED coating method offered uniform, conductive, 2D thin films covering over complex, flexible surface areas. This work was the first synthesis of 2D conductive MOFs as electrodes for flexible supercapacitors. The performance is outstanding as evidenced by the high specific areal capacitances (11.5 mF cm^−2^ and 22.1 mF cm^−2^ in aqueous and organic electrolytes, respectively), the high power density (1.35 mW cm^−2^ at 1 mA cm^−2^, organic system), and the energy density (22.4 μWh cm^−1^ at 0.1 mA cm^−2^, organic system).

Besides the Ni foam substrate, a stainless-steel surface has also been coated with Ni-MOF for supercapacitor applications. In one contribution, through solvothermal synthesis, Ni-BDC MOFs displayed an excellent performance with a specific capacitance of 850.42 F/g at a current density of 1 mA/cm^2^ with a maximum energy density of 18.66 Wh/kg and power density of 1671 W/kg.

In another contribution [85], Ni-Co MOFs composited with reduced graphene oxide (rGO) were formed as a film to improve its electron transfer property. Here, upon cleaning, nickel foam (NF) substrates were mixed with the Ni-Co MOF/rGO (prepared separately), polyvinylidene fluoride (PVDF), and activated carbon using a mortar and pestle to form a homogeneous slurry by adding dropwise N-Methyl-2-pyrrolidone (NMP). This is an elegant demonstration of coating MOF-based nanocomposites on substrates. The resultant nanocomposites show a high specific capacitance of 1320 F g^−1^ at 4 mA cm^−2^. The asymmetric activated carbon/Ni-Co MOF/rGO displays an energy density of 94.4 Wh kg^−1^ and a power density of 1291 W kg^−1^, with a 90.6% capacitance retention after 5000 cycles.

MOFs have also been coated onto flexible, wearable electronic textiles with supercapacitor applications. In one example [86], a bimetallic water-soluble MOF, UTSA-16 (Co/Zn), was coated on textiles through screen printing, pad-dry-coating, and inkjet print methods. Such a combination of coating approaches made it possible to expand the use of MOF coating to wearable, flexible textiles. All three methods are suitable for complex, flexible substrate coating with standalone MOFs. The resultant e-textiles show high supercapacitor performances, as evidenced by the high areal capacitance of ~221.51 mF cm^−2^, ~359.4 mF cm^−2^, and ~353.5 mF cm^−2^ at a scan rate of 1 mVs^−1^ for screen print, pad-dry-coating, and inkjet printing, respectively. The energy densities, ~123.06 µWh cm^−2^ (screen print), ~199.66 µWh cm^−2^ (coating), and ~196.39 µWh cm^−2^ (inkjet print), and the power densities, ~55,377.5 µW cm^−2^ (screen print), ~55,291.54 µW cm^−2^ (coating), and ~54,385.38 µW cm^−2^ (inkjet print), together with the high capacitance retention (~97.4–97.9%) after 1000 cycles, made them excellent supercapacitors for wearable electronic textiles.

### 3.3. Electrochemical Sensor

**Figure 13:**
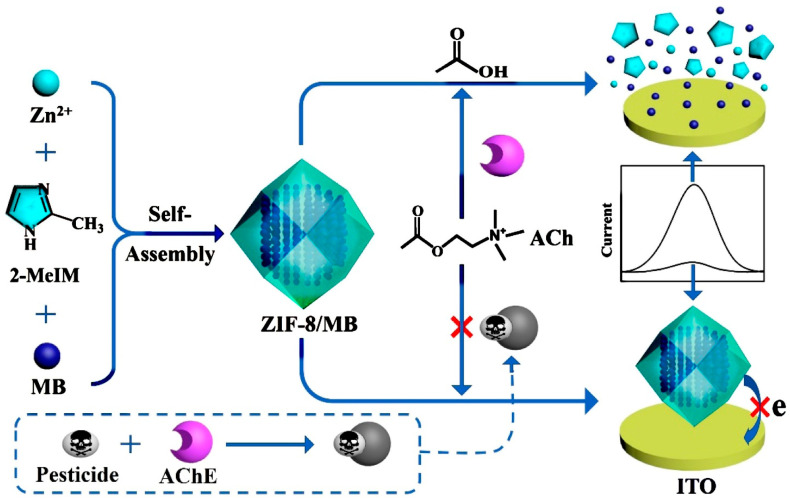
Representative illustration of the synthesis of ZIF-8/MB composites and the principle of the ZIF-8/MB composites-based electrochemical sensor for pesticide assay. Reproduced with permission from Ref. [87] Copyright: Sensors and Actuators B: Chemical, 323, 128701; 2020, Elsevier.

Electrochemical sensing is another major application of MOF-based coatings (Figure 13) [87]. In one contribution [88], presynthesized bimetallic MOFs, Mn/Fe-BDC MOFs, were suspended in 2-propanol with a Nafion polymer binding agent. Then, the classic drop-casting method was applied to coat the surface of a carbon working electrode of the screen-printed electrode (SPE) and dried under ambient temperature and pressure to form a uniform layer of coating (with a gentle wash to remove impurities or unbound materials). The MOF showed high adsorption and electrocatalytic properties towards chlorpyrifos due to the heterometal synergism arising between the Mn^2+^ and Fe^3+^. A square wave voltammetry-based electroanalysis was applied for the detection of chlorpyrifos with a remarkably low detection limit of 0.85 nM or 0.29 ppb in a wide linearity range of 1–100 nM with exceptional repeatability and anti-interference performance. Here, drop-casting was selected due to its simple operation and wide adaptability to various substrates. In another elegant contribution [89], a Co-based MOF, ZIF-67, was photothermally decomposed into a laser-induced graphitic (LIG) carbon electrode. Under laser, the MOF became small Co/Co_3_O_4_ core-shell nanoparticles (5–15 nm) uniformly distributed in the electrode region. The inclusion of MOF drops the impedance by 100 times compared to the traditional, polyimide-based sensor. It shows 400 times higher capacitance (2.4 mF cm^−2^). The MOF-derived composites offer higher specific surface areas and accessible electrocatalytic active sites, facilitating charge transfer and electrochemical reactions at the electrode−electrolyte interface. More applications of MOF-coating in flexible electrochemical sensing applications are anticipated due to dynamic responses to physical and chemical signals, as well as the development of coating techniques. A comprehensive review summarizes the applications of MOF films on wearable devices, environmental monitoring, and healthcare diagnostics [90].

### 3.4. Gas Sensor

**Figure 14:**
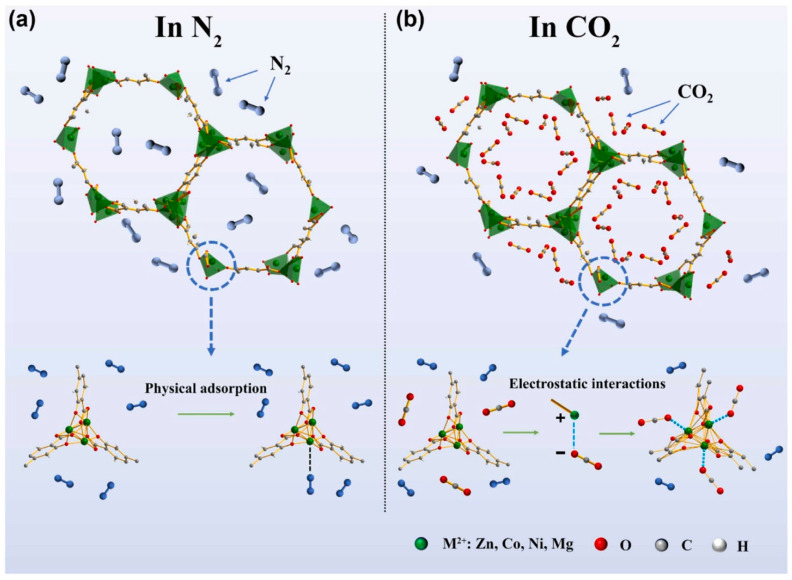
Representative illustration of the nanoscale M-MOF-74 (M = Mg, Ni, Zn, Co) based quartz crystal microbalance (QCM) gas sensor for the detection of CO_2_ gas at room temperature. As shown in (**a**), the M-MOF-74 sensor is in N_2_, there is minimal adsorption of N_2_ molecules. In (**b**), when M-MOF-74 QCM gas sensors are exposed to CO_2_ the open metal sites of the MOF-74 play a fundamental role in the CO_2_ adsorption properties. Reproduced with permission from Ref. [91]. Copyright: Sensors and Actuators B: Chemical, 413, 135874; 2024, Elsevier.

Gas sensing can also be achieved with MOF films/membranes (Figure 14) [91]. For example, in ref. [92], three MOF-74 (Co, Mg, and Ni as the metal ion precursors) were coated onto the surface of the glass/Pt interdigitated electrodes with a terminal -COOH for electrical detection of a toxic industrial acid gas, NO_2_. Both in situ growth and drop-casting coating methods were employed to prepare various needed electrodes for gas detection. Here, the interdigitated electrode (IDE) was either mixed with metal ions and ligands in water–DMF–ethanol mixing solvents, followed by heating to form a thin film on IDE, or pipetted with presynthesized MOF-74 drop-by-drop. The high surface area and tunable pore size/shape make MOFs useful materials for selective gas capture and sensing. The carboxylate groups allowed the three MOFs to grow relatively uniformly (differs slightly among metals) to form a thin film membrane of the Pt electrode. Ni-MOF-74, as the most consistently fabricated, thin and homogenous membrane, was exposed to 5 ppm NO_2_. The impedance dropped by 123 times in 4 h while the use of the MOF membranes as a sensor for NO_2_ enables continuous detection with overlapping growth. The selectivity of NO_2_ against interference gas, such as SO_2_, CO_2_, NO, and NH_3_, has been improved by the synthesis of a conductive bimetallic MOF, the CoCe-BTC [70]. This MOF was spin-coated on poly(3,4-ethylenedioxythiophene) (PEDOT) to prepare a composite film. Here, spin-coating was employed wherein CoCe-BTC was dissolved in ethanol. PEDOT aqueous dispersion was then dried and redispersed in ethanol. Next, a CoCe-BTC solution was dropped onto an IDE and spin-coated for five layers and dried. Finally, PEDOT was dropped onto the IDE to form an MOF-composite film. The spin-coating method offered simple and efficient preparation of the MOF coating in this application. The CoCe-BTC/PEDOT composite film is 1.2 times more sensitive to NO_2_ gas than pure PEDOT. The dynamic range is 5–50 ppm with good reproducibility. The presence of the MOF improves the conductivity of the conductive polymer, which causes the enhancement in NO_2_ sensing. The pore size/shape variety of MOFs has been further explored, together with conductive MOF films, to achieve the tunable electrical sensing of various gases. For example, in ref. [69], dual-MOF-layered films were prepared via a solution shearing-based film fabrication where a conductive MOF film was coated on a sensing substrate for signal detection, while a second layer of MOF film with different pore structures, adsorption properties, and thus, gas diffusivity was selectively integrated on top of the conductive MOF layer. The selected coating method allows for dual layer film formation, ideal for this application. The resultant dual-layer MOF membrane showed tunable sensing, enhanced sensitivity, selectivity, response speed, and recovery for analytes such as NH_3_, H_2_S, and NO_2_. For example, when ZIF-8 was coated on the conductive MOF layer, NH_3_ was able to pass through and be detected, yet H_2_S was sieved out. When MIL-53-TDC was coated on the conductive MOF layer, H_2_S was accumulated in and was sensitively detected.

### 3.5. Biosensor

**Figure 15:**
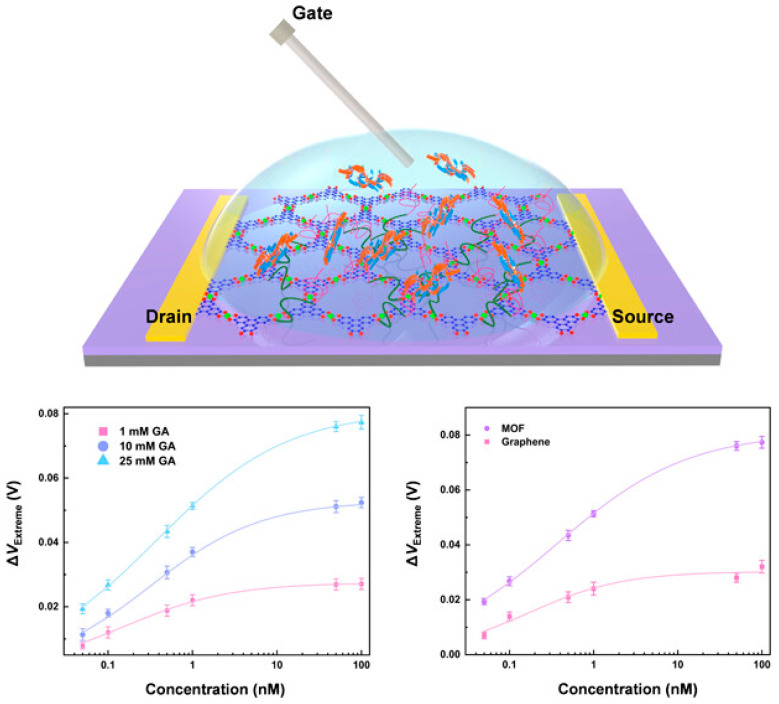
Representative illustration of the aptameric Ni_3_(HITP)_2_ MOF biosensor configured as a field-effect transistor for the detection of tumor necrosis factor α (TNF-α). The sensor exhibits 2.7 times higher responsivity and 34% of the limit of detection than a graphene-based counterpart. Reproduced with permission from Ref. [93]. Copyright: ACS Applied Nano Materials, 7(14), 17081–17091; 2024, ACS.

The sensing of bioactive molecules in native environments is also widely required in environmental, biological, and analytical sciences and industries (Figure 15) [93]. A typical biosensor based on MOF coating has been demonstrated by the fabrication of MOF films, the MIL-53(Fe), on a carbonized natural seaweed ZnO nanomembrane as an induction layer [94]. Here, the coating method, atomic layer deposition (ALD), was selected to ensure the control over film thickness and uniformity. The resultant device was applied as a dopamine biosensor based on potential detection via electrochemical approaches. Upon optimization of the geometry and structure of the composite and applied potential, the sensing performance was determined. The obtained sensor demonstrates ultra-high sensitivity (2084.58 μA mM^−1^ cm^−2^, a wide linear range, a low limit of detection (0.73 µM), and a good distinction between DA and ascorbic acid at an optimized potential of 0.3V.

Electrochemical biosensors represent one of the most prominent and impactful applications of MOF-coated electrodes. MOF coatings play multifaceted roles in electrochemical biosensors, particularly for biomedical and healthcare applications. Their structural tunability, porosity, and chemical versatility make them ideal for enhancing sensitivity, stability, and selectivity. In particular, MOFs enhance the sensitivity of biosensors due to their high surface area (which amplifies detection signals), electrocatalytic properties (which amplify electrochemical response even at low analyte concentrations in redox reactions), conductivity in certain cases (which facilitates fast electron transfer between the analyte and electrode, enhancing current response and reducing detection limits), and ability to hybridize with graphene, CNTs, or metal nanoparticles to show synergistic conductivity and sensitivity. MOFs enhance the selectivity of biosensors mainly due to their pore size tuning and molecular sieving capacity, ease of chemical functionalization (to select specific biomarkers), and capability to immobilize enzymes or antibodies (to prevent nonspecific adsorption). MOFs also enhance the stability of biosensors by protecting for enzymes and other biomolecules (shielding them from proteolytic, heat, or other adverse environments) while stabilizing delicate electrodes from fouling and physical wear.

### 3.6. Corrosion Resistance

**Figure 16:**
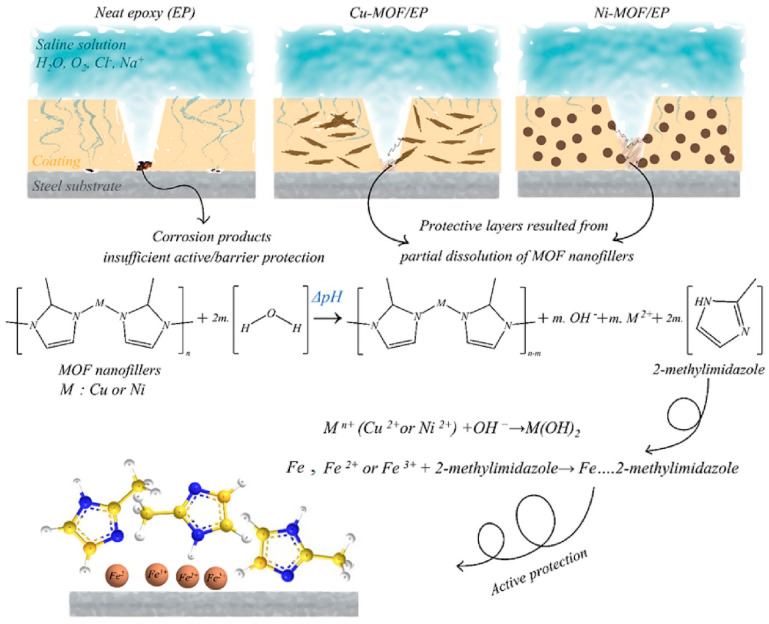
Illustration of the enhanced anti-corrosion mechanism of Cu-MOF/EP (epoxy) and Ni-MOF/EP nanocomposite coatings compared to neat EPs. Reproduced with permission from Ref. [95]. Copyright: Progress in Organic Coatings, 183, 107803; 2023, Elsevier.

A thin film layer of MOFs, in principle, can also be applied as corrosion-resistance coatings due to their wide variety of structures, porosity, electrical properties, and high stability. In one approach, leveraging the high porosity of MOFs, traditional corrosion inhibitors can be entrapped within MOFs via a brushing process. Under corrosive conditions, corrosion inhibitors can be released from the MOF to protect the substrate surfaces (Figure 16) [95]. Alternatively, MOFs can serve as an isolation layer of superhydrophobic anticorrosion coatings to separate the substrate surface from corrosive resources. Recently, the classic hydrothermal method to synthesize MOFs has been bypassed by an induced electrodeposition of anticorrosion MOFs on a porous oxide film tightly mounted on the aluminum alloy [25]. In doing so, Ce(NO_3_)_3_ and H_3_BTC precursor solutions were used as the electrodeposition liquid in the electrolytic cell, a porous anodic alumina was used as the cathode, and a graphite electrode was used as the anode. A brushing process was then carried out to prepare the composite coating. A voltage of 20 V was applied to deposit the MOF onto the alumina at room temperature (25 °C) for 2 h, followed by rinsing and drying of the deposited coatings. The advantages of electrodeposition, such as the precise control over film thickness and the compatibility with electronic devices, were employed for this special application. The resultant coating layers were tested, which found that the resistance was three orders of magnitude higher than that of the aluminum alloy alone. The corrosion current density is three orders of magnitude lower than that of the aluminum alloy alone at 1.621 × 10^−8^ A/cm^−2^. The corrosion resistance efficiency is 99.92%. The salt spray experiment shows that the film can maintain corrosion resistance for 30 days. In another elegant contribution, water-stable Zr-MOFs were combined with epoxy to prepare a composite film on the surface of cold-rolled steel (CRS) [96]. A curing resin, trimethylolpropane tris[poly(propylene glycol) and amine-terminated] ether, was introduced to ensure the formation of uniform slurries of Zr-BTB/epoxy composites. The slurry was then applied onto CRS at 120 °C for 5 h. The resistance was found to be 197.32 kΩ·cm^2^ after 24 h of immersion in NaCl solution, in contrast to the resistance of 26.22 kΩ·cm^2^ of the epoxy coating alone. Blockage of corrosive ion penetration was found to be effective from a 480-h salt spray test. The coatings also showed low oxygen permeability in comparison to epoxy coating alone, likely due to the high energy barrier and aspect ratio inherent to the 2D MOFs.

### 3.7. Biomedical Applications

**Figure 17:**
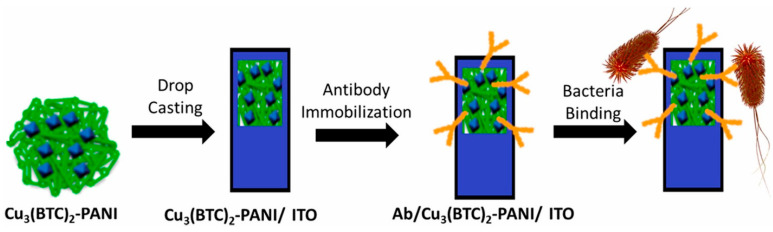
Representative illustration of procedure for bioelectrode fabrication and its subsequent utilization for impedimetric sensing of *E. coli*. Reproduced with permission from Ref. [98]. Copyright: Environmental research, 171, 395–402; 2019, Elsevier.

MOFs have also been widely applied in biomedicine [97]. Of particular interest is the MOF composite-based coating, which has been reported in several cases (Figure 17) [98]. For example, in ref. [99], polydopamine-modified (PDA) ZIF-8 (PZIF-8) particles were applied as fillers for polycaprolactone (PCL) coatings on Mg alloys without changing the structure and crystallinity of the MOF. The inclusion of PZIF-8 into the PCL coating increased the surface roughness while strengthening its bonding with the substrate. PZIF-8 also offers enhanced corrosion resistance and bioactivity with improved cell adhesion, highlighting the benefits of PZIF-8 PCL composites as coatings for Mg-based orthopedic implants. MOFs have also been employed as biocompatible coatings to enhance the bioactivity and antibacterial properties of another substrate, Ti6Al4V [99,100]. In doing so, gallic acid as a natural organic linker and Zn^2+^ as the metal ion precursor were employed to form an MOF structure. The Ti6Al4V substrate was treated with NaOH, followed by the addition of the MOF precursors. The surface of the metal was covered with a uniform layer of Zn-MOFs to accelerate mineralization with superior antibacterial properties compared to Ti substrates alone. The synthesis was carried out via an in situ growth hydrothermal method, wherein titanium discs were coated by adding gallic acid and Zn(NO_3_)_2_ to form the needed film. In another recent work, bimetallic Mg/Cu-MOFs were coated on Zn membranes in order to enhance their biological performance for bone regeneration [100]. Here, a facile one-step hydrothermal method was employed to promote the growth of Mg/Cu-MOF coatings on zinc discs. The selected coating method in both cases is suitable for complex device coating. The Zn^2+^ released from the membrane, together with the Mg^2+^ and Cu^2+^ from the MOFs, displayed a synergistic effect on speeding up the deposition of a calcium phosphate layer, enhancing osteoblast proliferation, vascularization, and antibacterial efficacy against *S. aureus* and *E. coli*.

In addition to the recent representative applications of MOF coatings discussed above, an increasing number of research and industrial areas could, in principle, also emerge or be integrated. These include the environmental monitoring of toxic gases or liquids beyond those discussed above, electrocatalytic processes, such as the oxygen reduction and evolution reactions (ORR/OER), CO_2_ reduction, and hydrogen evolution reactions (HER), all of which will benefit from the tunable, highly porous, and electrical properties of MOFs. In addition, MOF coatings have been—and can be explored—in photovoltaics and optoelectronics as dye-sensitized solar cells (DSSC), photodetectors, and OLEDs. Other promising directions include gas separation membranes, water purification and desalination systems, and controlled-release coatings, underscoring the versatility and expanding relevance of these materials. Interested readers are referred to more application-focused review articles published recently.

### 3.8. Heat Exchangers

One of the most significantly impacted and benefited areas from the innovation of MOFs and MOF-based nanocomposites is the heat exchanger [101]. The exceptional properties of MOFs and associated nanocomposites, such as high surface area, finely tunable porosity, and adsorption capabilities, make them ideal additions to current heat exchangers. The current heating, ventilation, and air conditioning (HVAC) and related systems have shown improved heat transfer efficiency, moisture control, and energy efficiency due to the incorporation of MOFs and associated nanocomposites. These advancements would not have been possible without recent advances in MOF synthesis techniques, which ensure the scalability for industrial applications, as well as surface functionalization and coating methods, which allow for MOFs and associated nanocomposites to be coated on heat exchanger surfaces (especially fins with larger surface areas in various shapes) with high thermal and mechanical stability [101]. Since the design and working principles of HVAC systems involving heat exchangers have been well-known, here we only focus on how MOF composite coatings can improve heat exchangers’ performances. Our discussion will be centered on how MOF composites impact thermal conductivity, fouling resistance, material degradation (corrosion), and heat exchanger design.

Thermal conductivity is critical to the performance of a heat exchanger as it determines the heat transfer efficiency, required surface area, temperature uniformity, and transient response. Cu and Al are usually the choice of materials for heat exchangers due to their high thermal conductivity. However, their high thermal expansion and degradation rate under harsh conditions result in enhanced thermal resistance. MOFs and associated nanocomposites can overcome these barriers by reducing interfacial thermal resistance at the nanoscale. The principle is due to the improved phonon transport through the ordered pathways in MOF-polymer composites that reduce scattering events at the material interfaces. A number of MOFs, such as MIL-101, UiO-66, and HKUST-1, have been incorporated into heat exchanger materials and have shown enhanced thermal conductivity [102,103,104]. Key methods to incorporate MOFs into metal and polymer matrices for heat exchanger applications include (i) in situ growth, which offers strong interfacial adhesion and uniform coating [105], (ii) physical blending, which offers high cost efficiency and scalability but suffers from uniformity and interfacial bonding [106], (iii) electrochemical deposition, which is more suitable for metal matrices and offers precise control over composite microstructure and excellent interfacial adhesion [37], and (iv) surface functionalization [107], which offers enhanced compatibility and bonding between MOFs and matrices, and thus reduces thermal resistance and optimizes thermal transport.

Because most heat exchangers are installed in an environment fully exposed to open air or water/fluids (for heat release obviously), their contact with dust, biological species, and mineral deposits is inevitable. Such contact can result in a significant decrease in heat exchange rates and an increase in energy consumption. Of particular importance is fouling, which, due to the growth of microbial species, can form biofilms which block flow channels, reduce membrane permeability, and increase thermal resistance, placing a high demand on antifouling coatings and self-cleaning heat exchanger materials. The tunable pore sizes, high surface area, and selectivity against different adsorbents of MOFs and associated nanocomposites endorse them as excellent anti-fouling components for heat exchangers. Hydrophobic MOFs can help isolate condenser unit materials from water and other contaminants in water, preventing moisture and associated biofilm formation. Typical MOF-based composites employed for antifouling applications include ZIF-8 or HKUST-1@PVDF45, MIL-110, CuZn-MOF-74 (PBMA), GO/ZIF-8, and PDMS@MOF@Cu [108,109,110,111,112]. The last one even exhibits self-cleaning properties [112].

Material degradation, especially corrosion, can also impact the performance of heat exchangers, particularly when corrosive environments and/or high temperatures are encountered. Corrosion can involve complex mechanisms; in addition to the resistance against biofouling-induced corrosion (see the discussions above), MOFs, such as MIL-101(Cr) or UiO-66, in combination with Al materials, exhibit increased resistance to corrosion and high-temperature degradation [113], likely due to the high surface area and porosity of the involved MOFs. This results in faster heat dissipation, which in turn enhances the stability and longevity of the heat exchanger materials.

In terms of heat exchanger designs that involve MOF and associated nanocomposites, attention has been paid to compact plate heat exchangers (PHEs), fin tube heat exchangers, and wire-finned tube heat exchangers. For PHEs, ZIF-8/PI composite-coated plates showed enhanced heat transfer rates while reducing fouling. Other MOFs, such as Al-fumarate, CAU-23, CAU-10, and MIL-100 (Fe), also demonstrated improvement in antifouling and energy reduction performance when applied in PHEs [114,115]. In high temperature/pressure conditions, combining MOFs with fin tube heat exchangers offers reduced thermal resistance, enhanced fluid flow across the heat exchanger surfaces, effective heat dissipation, and excellent mechanical stability. MIL-160 (Al), MIL-100 (Fe), and Al-fumarate have been explored in this direction [116,117]. In HAVC, especially refrigeration systems, MOFs incorporated with wire-finned tube heat exchangers promote their performances due to MOFs’ moisture adsorption and desorption/dehumidification capabilities. Typical involved MOFs include MIL-100 (Fe), MIL-100 (Cr), and MOF-801, to name a few [118,119].

Besides the excitement of advancement provided by MOF and associated nanocomposite coatings in heat exchangers, there is still room to improve in order to fully adapt MOFs and associated nanocomposites in heat exchanger industry, such as enhancing the cost-efficiency, long-term stability and strength, enabling multiple functions in MOFs, developing green MOF coating approach, and fostering research–industrial collaborations.

### 3.9. Sustainable Scale Resistance

Another important application of MOF-based coating materials is resisting scale deposition, the buildup of CaCO_3_, Mg(OH)_2_, BaSO_4_, or silica, in various industries, such as power generation, oilfield and gas/chemical pipelines, food and beverage, paper, pharmaceutics, and seawater desalination [120]. Scale deposition typically reduces efficiency, increases operational costs, shortens equipment lifetime, and/or present safety and contamination risks. While classic scale resistance approaches possess various drawbacks, a recently developed, light-responsive MOF-based liquid-like coating showed exceptional anti-scale deposition performance. Herein, a stable, liquid-like surface coating was synthesized by encapsulating poly-spiropyran (PSP) into a functionalized MOF, which was later covalently connected with polydimethylsiloxane (LPDMS), forming the MPL coating [120]. The MOF pores/chambers prevent PSP aggregation, which helps preserve high ion-adsorption capacity upon illumination. Such an MPL coating demonstrated reversible anti-scaling performance under sustainable light-triggered control, a smart and adaptive solution to prevent mineral scaling [120]. The MOF scaffolds ensure the well-dispersed and effective functional groups, making them ideal for environments where controlled scaling is crucial—water treatment systems, smart filters, and industrial pipelines.

## 4. Limitations and Future Perspectives

### 4.1. Challenges and Limitations

Besides the significant potential of MOF-based coatings in solving real life problems across various areas, certain limitations or drawbacks are still present, hindering the broader applications and practical scalability of MOF-based coatings. The most significant issue is the stability and durability of MOF-based coatings. In the long run, a number of MOFs have been found to possess limited chemical, thermal, and mechanical stability. This is especially severe when MOF coatings are exposed to harsh conditions or environments, such as high humidity, extreme temperatures, high salinity, mechanical stress, or corrosive matter. Such vulnerability often reduces the long-term performance of MOF-based coatings while restricting their use in demanding industries. There have been strategies or efforts to enhance the stability of MOF-based coatings, such as post-synthetic modifications, incorporation with more robust materials to form composite films, and development or employment of intrinsically stable MOFs. There has been progress reported in these directions, yet consistent success remains elusive for different applications.

Another continuously occurring issue in MOF coatings is the uniformity and reproducibility of the resultant 2D MOF films. The preparation of homogeneous layers with controlled thickness, uniformity, and morphology remains technically challenging, particularly when scaling up from laboratory- to industrial-scale fabrication. Most current coating methods display batch-to-batch variability and require fine-tuned processing conditions, which complicate their integration into manufacturing pipelines. Additionally, the scalability and cost-effectiveness of these fabrication methods remain nontrivial barriers. Many MOFs also require complex synthesis procedures or expensive precursors, raising demands on the financial side.

Moreover, as MOF coatings become an attractive strategy in environmental and biomedical applications, their environmental impact should also be considered. The toxicity of certain metal ions or organic linkers has raised concerns about environmental and sustainability factors. Protocols for safe disassembly, disposal, and recycling are also under development, likely due to the lack of comprehensive life-cycle assessments. Innovative material design, scalable synthesis, and environmental engineering will be essential to address these concerns and unlock the full potential of MOF-based coatings.

There are also practical barriers to the scaling-up of MOF coatings. For example, for MOFs that require solvothermal or hydrothermal synthesis using toxic solvents (DMF, DEF) at elevated temperatures or pressures, it is challenging to scale up. Achieving consistent, reproducible particle size, morphology, and phase purity at large scale is difficult. It is also quite common for long synthesis times, challenging high-throughput manufacturing. The coating uniformity over large or irregular substrates are always concerns while special surface functionalization can add extra cost and processing steps. Solvent recovery and safe disposal may require additional infrastructure and cost. Tight environmental control can be needed as humidity and temperature can affect film quality. Reproducibility can be challenging as well due to the batch-to-batch variability, defects in MOFs (missing linkers or metal nodes), and variability in functionalization and posttreatment. Overall, in comparison to conventional coatings, MOF coatings are costly in terms of materials and processing and more difficult to scale up, especially for industry and field applications. However, they offer superior performance per unit cost (selectivity and tunability) and excellent customization opportunities. These factors all impact the industrial readiness of MOF-based coatings. For example, to improve industrial readiness, it is critical to reduce the cost and environmental impact in MOF synthesis, enhance adhesion, increase the mechanical robustness and flexibility of MOF coatings, and enhance continuous manufacturing (such as spray- and dip-coating).

### 4.2. Biocompatibility of MOF-Based Coatings

The broad applications of MOF-based coatings, especially in biomedical, biological, and environmental fields, raise concerns regarding biosafety and biocompatibility. In comparison to bulk MOF materials, MOF-based coating materials directly contact biological tissues, fluids, living organisms, and ecosystems, sometimes for extended durations. Thus, factors/consequences, such as the cytotoxicity, immune response, degradation behavior, and metal ion leaching of MOFs, whether from intentional or inevitable degradation upon contact the target media, need to be considered during the design and development of MOF-based coatings for relevant devices.

Key factors influencing biosafety and biocompatibility include the MOF composition, particle size and morphology, and MOF coating functionalization with polymers and metals. Metal center selection is not only critical for coordination stability but essential for minimizing cytotoxicity; a proper MOF coating should balance these two factors by using non-toxic metal ions that can provide sufficiently stable MOFs. The organic linker can also negatively impact cellular health due to its hydrophobicity/hydrophilicity, functional groups, and (by)products upon MOF degradation/damage. Many MOF-based composites are on the nanoscale to microscale, which may impact cell adhesion, uptake, and inflammation differently. Thus, a balance of functionality and cellular uptake should be met for a proper coating. As discussed in various places above, surface chemistry and functionalization are often combined with MOF materials in coating applications. Thus, biocompatible polymeric materials, amino acids, or biomolecules are often incorporated to enhance the biocompatibility of the resultant MOF-based composite coatings.

Although the “principles” to improve the biocompatibility of MOF-based coatings are relatively straightforward to understand, practically, it is challenging to meet these goals. Although many in vitro and in vivo assays are available for testing cytotoxicity, there is a lack of standardized testing protocols for MOF-based coatings, likely due to the high diversity in both MOF compositions and coating substrates, as well as the long duration required to fully understand the biocompatibility of an MOF coating. It is also common to see discrepancies between in vitro and in vivo testing results. There are also regulatory barriers and translational limitations when applying the developed devices. Lastly, there is an urgent need to safely degrade MOF coatings from devices when they are no longer needed. Smart MOF coatings, stimuli-responsive biocompatibility, and long-term studies are required to overcome these barriers. Interdisciplinary collaborations among scientists in various fields, such as materials science, biotechnology, medicine, and chemistry, are essential to further advance this field.

### 4.3. Future Perspectives and Research Directions

Looking forward, the future of MOF-based coatings will benefit from several transformative advances that could significantly enhance their performance, accessibility, and real-world impact. One exciting direction is the development of green synthesis and environmentally benign coating strategies. Eco-friendly fabrication routes in line with the principles of green chemistry can be achieved by minimizing the use of toxic solvents, reducing energy consumption, and employing sustainable precursors. These approaches will not only reduce environmental impact but improve the feasibility of large-scale production. For example, solvent-free or aqueous MOF synthesis, including mechanochemical synthesis, vapor-assisted conversion, solid synthesis, and aqueous phase MOF synthesis, can remove concerns about solvent toxicity. The synthesis temperature should also be reduced to near room temperature to lower energy costs. Ultrasound-assisted synthesis in the aqueous phase may also be employed to replace the current high temperature/pressure synthesis. Eco-friendly coating techniques, such as dip-coating, spray-coating, spin-coating, and in situ growth on substrates can also improve the green preparation of MOF coatings, as long as water or minimal amounts of ethanol are employed to disperse MOFs. If needed, these eco-friendly coating approaches can be combined with a hydrothermal method to enhance adhesion. Lastly, certain green additives or functionalization species can be included to enhance the green level of an MOF coating. For example, biodegradable binders can be employed for adhesion. Natural templates, such as starch or silk, can guide coating morphology. Enzymes or other bioactive molecules can be incorporated to add functionality and sustainability in various applications.

Another frontier is the integration of computational tools, such as machine learning, molecular simulations, and high-throughput screening, to guide the rational design of MOF structures and to predict their coating behavior under specific conditions. These tools will enable the rapid identification of optimal materials and processing parameters, greatly speeding up the pace of discovery and reducing experimental trial-and-error. In parallel, the development of multifunctional MOF-based composite coatings by combining MOFs with polymers, nanoparticles, or other robust matrices will open new avenues for advanced coating performance. These composite materials/coatings/films will deliver synergistic properties, such as enhanced stability, selective permeability, or tailored responsiveness, to external stimuli, broadening their application spectrum even more.

Finally, translating the technical innovations from lab benches to industry remains challenging. Preparing MOF coatings with industrial scalability requires not only the refinement of cost-effective synthesis and coating techniques but the standardization of quality control and integration into existing production approaches. Overcoming these barriers will be key to realizing the commercial potential of MOF coatings in industrial sectors such as energy, electronics, healthcare, and environmental technology. Looking further into the future of MOF coatings, applications in smart coatings for adaptive surfaces, wearable sensors, flexible optoelectronic devices, and targeted therapeutic delivery systems will make the best use of the vast potential of MOF-based coatings. With continued interdisciplinary collaboration and technological advancement, MOF coatings are well-positioned to become a cornerstone of the next generation of advanced functional materials. Note that these directions and gaps are only based on our understanding and interests in MOF coatings’ applications. The direction and gaps can easily be different from different personnel with varied perspectives and viewpoints.

## 5. Conclusions

In conclusion, we summarized the most recent findings in experimental methods that can coat MOF materials onto the surfaces of metallic electrodes, polymeric thin films, and functional glass, with a focus on the key requirements and advantages of each coating method and the applicable substrates. Although the applications of coating MOFs on metallic electrodes, polymeric thin films, and functional glass can in principle be massive, we highlighted a few areas where MOF coatings made significant contributions, especially those reported in the past 3–5 years, which would also stimulate broader interest in MOF coating techniques and materials. Lastly, the current bottlenecks and limitations of MOF coatings on the three material surfaces discussed in this article have been summarized, namely stability, crystallinity, and fine controls in coating thickness and properties. Future efforts of MOF coatings should focus on overcoming these challenges, together with making MOF coatings more environmentally friendly and biocompatible, as well as scalable for industrial applications.

## Figures and Tables

**Table 1:** Comparative Summary of Methods for Coating MOFs on Substrates.

Coating Method	Principle of Operation	Advantages	Limitations	Coated MOFs	Substrates	Key Parameters/Consideration	Refs.
Anodic electro-deposition	Oxidation at the anode releases metal ions to react with ligands in solution and form MOFs on anode surface.	Controlled growth; tunable thickness; compatible with electronic devices.	Limited to conductive, sacrificial anode materials.	Cu-BTC; Ce-MOFs; Ni-MOF-74; HKUST-1 MOF.	Electrodes; metal surfaces.	The voltage applied varies by metal.	[24,25,26,27,28,29,30,31]
Cathodic electro-deposition	Reduction at the cathode generates OH^−^ to increase pH and to trigger ligand deprotonation; metal ions in solution then react with deprotonated ligands to form MOFs on the cathode.	Better control over MOF composition; applicable to non-conductive substrates including thin films and glass.	Side-reactions may occur if pH is not well-controlled; adhesion may vary.	ZIF-8; ZIF-67; Co-MOF; Fe/Co-MOF; MOF-5; MIL-100.	Electrodes; inert polymeric thin films; non-conductive glass.	Voltage applied is ~1.5 V; metal ion concentration needs to be regulated in solution.	[24,32,33,34,35,36,37,38,39]
Electro-phoretic deposition	Charged, presynthesized MOFs are immobilized onto an oppositely charged electrode surface under an electric field.	Simple operation; universal applicability.	Film/coating stability may require extra design: poor uniformity if MOFs suspension is unstable.	UiO-66; UiO-66-NH_2_; Ni-MOF-74.	Negatively charged surfaces, such as the FTO glass.	It requires stable MOF suspension as well as control over voltage and deposition time.	[26,40]
Solvothermal /hydrothermal in situ growth	Metal ions and ligands react in organic solvents or water at high temperature and pressure to grow MOFs directly on the immersed substrate surfaces.	Strong adhesion; uniform coating; high crystallinity.	MOFs and substrates sensitive to temperature are not adaptable; reaction rate is slow; it often uses toxic solvents; it is difficult to control thickness/morphology.	Cu-BTC; Ni-MOF; Ni/Co MOFs; multi-variant MOFs.	FTO; metal films (Cu, Ni); stainless steel; glass; stable polymers.	Critical surface cleaning; pretreatment (plasma etching, salinization); control of precursor concentration, temperature, and duration; modulators are needed.	[41,42,43,44,45,46,47,48,49,50]
Layer-by-Layer (LbL) deposition	Sequential deposition of precursor layers, such as metal ions and organic linkers, followed by rinsing steps builds up one MOF layer at a time.	Atomic level thickness control; exceptional uniformity; tunable composition; formation of heteroepitaxial interfaces.	It is time-consuming for thick films; it may require vacuum and volatile precursors; it is limited by precursor suitability and film stability.	Zn-BPDC; Cu-BDC; PDI-linker MOFs.	Silicon; glass; FTO; liquid gallium.	Alternating precursor exposure and rinsing; proper substrate pretreatment and temperature control.	[20,51,52,53,54,55,56,57,58,59,60,61,62,63,64,65]
Vapor Phase Deposition: vapor-assisted conversion (VAC)	Evaporated precursors react with solvent vapor to form crystalline MOF films.	Highly crystalline films; mild reaction conditions.	It requires a solvent; there is a potential for corrosion, contaminant, or non-uniform coating.	ZIF-8; ZIF-67, PCN-6; CAT-1; Co-MOF-74; Ni-MOF-74; Mg-MOF-74; PCN-222; UiO series.	Compatible precursors.	Controlled precursors and solvent ratio; moderate temperature.	[66,67]
Vapor Phase Deposition: vapor-phase transformation (VPT)	Metal oxide precursor film is transformed into MOF film by reaction with vapor-phase organic linkers.	Solvent-free; superior shape retention and controllability.	Demanding conditions; thickness deformation; reduced mechanical stability.	ZIF-8; ZIF-61; ZIF-67; ZIF-72; Cu-CDC; Cu-BDC; HKUST-1.	Substrates precoated with metal oxide films.	CVD/PVD for oxide film; controlled organic linker vapor exposure.	
Vapor Phase Deposition: vapor-phase linker exchange (VPLE)	Existing MOF or hybrid film reacts with organic linker vapor to exchange linkers.	Allows property modification; minimal film deformation.	Mechanism or degree of exchange needs more study.	ZIF-8; ZIF-8/I; ZIF-8/Br; carboxy-late MOFs.	Substrates with preexisting MOF/hybrid films.	Controlled exposure to linker vapor.	
Vapor Phase Deposition: atomic layer deposition (ALD)/ molecular layer deposition (MLD)	Sequential exposure of substrate to volatile precursors in the vapor phase to form MOFs.	Atomic level thickness control; exceptional uniformity; tunable composition; formation of heteroepitaxial interfaces.	Challenges in crystallinity; a limited range of MOFs; it requires volatile/stable precursors.	MOF-5 nanofilm; NU-1000; UiO-66; ZIF-8, Cu-TPA; Mn; Co-based MOF films.	Various, suitable for vacuum.	Controlled precursor pulses; temperature cycles.	[65]
Spin-coating	MOF solution or slurry is spread on a rotating substrate by centrifugal force while solvent evaporating.	Simple; rapid; scalable; cost-effective; good uniformity; can be combined with LPE.	Limited surface size/curvature; high viscosity; spin rate control are needed.	Cu_2_(bdc)_2_•xH_2_O; Zn_2_(bdc)_2_•xH_2_O; HKUST1; ZIF8.	Gold; silicon; glass; porous stainless steel; aluminum oxide.	Substrate preprocessing; control over spin speed/time and solution viscosity.	[55,68,69,70,71,72]
Dip-coating	Substrates are dipped into an MOF solution or suspension and withdrawn at a controlled rate.	Simple; scalable; applicable to complex shapes.	Inconsistent quality (gravimetric effects); thermal stability of substrate during drying.	UiO-67; Cu-HHTP MOFs.	FTO glass; polymer sheets.	Controlled withdrawal speed; solution stability; drying conditions.	[73,74,75]
Spray-coating	Fine droplets of MOF solution or suspension are sprayed onto the substrate.	Rapid; suitable for large or complex surfaces; can be combined with LbL or LPE.	Uniformity depends on technique; potential material waste.	UiO-66; Zn-TPE; Cu3(HHTP)2.	Glass; metals; plastics; quartz; sapphire; Si/SiO_2_.	Control over spray pressure, distance, and nozzle; substrate temperature; precursor solution stability.	[76,77,78,79]
Drop-casting	Droplets of MOF solution or suspension are placed on the surface and dried.	Easy operation; coats complex materials.	Difficult to control thickness or uniformity; potential cracking; small scale.	UiO-67; Mn/Fe-BDC MOFs.	Polymer sheets; screen-printed electrodes.	Solution concentration or volume; drying conditions; surface wettability.	[74,75,76,77,78,79,80]

## Data Availability

All data and figures presented here received official copyrights from the original publisher.

## References

[1] Deng H., Grunder S., Cordova K.E., Valente C., Furukawa H., Hmadeh M., Gándara F., Whalley A.C., Liu Z., Asahina S. (2012). Large-Pore Apertures in a Series of Metal-Organic Frameworks. Science.

[2] Furukawa H., Cordova K.E., O’Keeffe M., Yaghi O.M. (2013). The Chemistry and Applications of Metal-Organic Frameworks. Science.

[3] Zhou H.-C.J., Kitagawa S. (2014). Metal–Organic Frameworks (MOFs). Chem. Soc. Rev..

[4] Du Y., Jia X., Zhong L., Jiao Y., Zhang Z., Wang Z., Feng Y., Bilal M., Cui J., Jia S. (2022). Metal-organic frameworks with different dimensionalities: An ideal host platform for enzyme@MOF composites. Coord. Chem. Rev..

[5] Fang Y., Powell J.A., Li E., Wang Q., Perry Z., Kirchon A., Yang X., Xiao Z., Zhu C., Zhang L. (2019). Catalytic reactions within the cavity of coordination cages. Chem. Soc. Rev..

[6] Kirchon A., Feng L., Drake H.F., Joseph E.A., Zhou H.-C. (2018). From fundamentals to applications: A toolbox for robust and multifunctional MOF materials. Chem. Soc. Rev..

[7] Lian X., Fang Y., Joseph E., Wang Q., Li J., Banerjee S., Lollar C., Wang X., Zhou H.-C. (2017). Enzyme-MOF (metal-organic framework) composites. Chem. Soc. Rev..

[8] Wang X., Lan P.C., Ma S. (2020). Metal-Organic Frameworks for Enzyme Immobilization: Beyond Host Matrix Materials. ACS Cent. Sci..

[9] Ding M., Cai X., Jiang H.-L. (2019). Improving MOF stability: Approaches and applications. Chem. Sci..

[10] Freund R., Zaremba O., Arnauts G., Ameloot R., Skorupskii G., Dincă M., Bavykina A., Gascon J., Ejsmont A., Goscianska J. (2021). The Current Status of MOF and COF Applications. Angew. Chem. Int. Ed..

[11] Horcajada P., Gref R., Baati T., Allan P.K., Maurin G., Couvreur P., Férey G., Morris R.E., Serre C. (2012). Metal–Organic Frameworks in Biomedicine. Chem. Rev..

[12] Shi R., Yu Y.-X., Chibisov A.N. (2025). Electrochemical reduction of cyanide on conjugated copper-organic framework Cu_3_(HHTP)_2_ monolayer: A dispersion-corrected DFT investigation. Int. J. Hydrogen Energy.

[13] Liu Y., Zhao Z., Li M., Zhao Z. (2024). Metal–organic framework thin films: Review of their room-temperature synthesis and applications. J. Mater. Chem. C.

[14] Sabzehmeidani M.M., Gafari S., Jamali S., Kazemzad M. (2024). Concepts, fabrication and applications of MOF thin films in optoelectronics: A review. Appl. Mater. Today.

[15] Jeong H., Park G., Jeon J., Park S.S. (2024). Fabricating Large-Area Thin Films of 2D Conductive Metal–Organic Frameworks. Acc. Chem. Res..

[16] Peng Y., Xu J., Xu J., Ma J., Bai Y., Cao S., Zhang S., Pang H. (2022). Metal-organic framework (MOF) composites as promising materials for energy storage applications. Adv. Colloid Interface Sci..

[17] Shen M., Ma H. (2022). Metal-organic frameworks (MOFs) and their derivative as electrode materials for lithium-ion batteries. Coord. Chem. Rev..

[18] Zhou C., Pan M., Li S., Sun Y., Zhang H., Luo X., Liu Y., Zeng H. (2022). Metal organic frameworks (MOFs) as multifunctional nanoplatform for anticorrosion surfaces and coatings. Adv. Colloid Interface Sci..

[19] Choudhary P., Ola S.K., Chopra I., Dhayal V., Shekhawat S.D. (2023). Metal–organic framework (MOF)/graphene–oxide (GO) nanocomposites materials: A potential formulation for anti-corrosive coatings- a review. Mater. Today Proc..

[20] Chen D.-H., Gliemann H., Wöll C. (2023). Layer-by-layer assembly of metal-organic framework thin films: Fabrication and advanced applications. Chem. Phys. Rev..

[21] Zhou Z., Xu L., Ding Y., Xiao H., Shi Q., Li X., Li A., Fang G. (2023). Atomic layer deposition meets metal–organic frameworks. Prog. Mater. Sci..

[22] Babu A.M., Varghese A. (2023). Electrochemical deposition for metal organic Frameworks: Advanced Energy, Catalysis, sensing and separation applications. J. Electroanal. Chem..

[23] Ulaeto S.B., Ravi R.P., Udoh I.I., Mathew G.M., Rajan T.P. (2023). Polymer-Based Coating for Steel Protection, Highlighting Metal–Organic Framework as Functional Actives: A Review. Corros. Mater. Degrad..

[24] Stassen I., Styles M., Van Assche T., Campagnol N., Fransaer J., Denayer J., Tan J.-C., Falcaro P., De Vos D., Ameloot R. (2015). Electrochemical Film Deposition of the Zirconium Metal–Organic Framework UiO-66 and Application in a Miniaturized Sorbent Trap. Chem. Mater..

[25] Cao Y., Wang L., Lu S., Wen Y., Shang W. (2023). Construction of porous anodic oxide/Ce-MOFs film by induced electrodeposition and its corrosion resistance. J. Ind. Eng. Chem..

[26] Borralho D., Realista S., Nunes T., Jorge M.E.M., Martinho P.N. (2024). Immobilisation of Ni(II) and Zr(IV) metal-organic frameworks on electrodes using electrophoretic deposition. ChemRxiv.

[27] Ito T., Jenkins S.G., Seifert S., Uysal A. (2023). Electrochemistry-Induced Direct Deposition of Nanoscale Thin Zeolitic Imidazolate Framework-8 Films on Insulator Substrates. Cryst. Growth Des..

[28] Van Assche T.R.C., Desmet G., Ameloot R., De Vos D.E., Terryn H., Denayer J.F.M. (2012). Electrochemical synthesis of thin HKUST-1 layers on copper mesh. Microporous Mesoporous Mater..

[29] Joaristi A.M., Juan-Alcañiz J., Serra-Crespo P., Kapteijn F., Gascon J. (2012). Electrochemical Synthesis of Some Archetypical Zn^2+^, Cu^2+^, and Al^3+^ Metal Organic Frameworks. Cryst. Growth Des..

[30] Campagnol N., Van Assche T.R.C., Li M., Stappers L., Dincă M., Denayer J.F.M., Binnemans K., De Vos D.E., Fransaer J. (2016). On the electrochemical deposition of metal–organic frameworks. J. Mater. Chem. A.

[31] Zhang X., Luo J., Tang P., Ye X., Peng X., Tang H., Sun S.-G., Fransaer J. (2017). A universal strategy for metal oxide anchored and binder-free carbon matrix electrode: A supercapacitor case with superior rate performance and high mass loading. Nano Energy.

[32] Bailmare D.B., Malozyomov B.V., Deshmukh A.D. (2024). Electrodeposition of porous metal-organic frameworks for efficient charge storage. Commun. Chem..

[33] Yan C., Shang J., Wan S.-Y., Cui X.-T., Liu Z.-G., Guo Z. (2025). In-situ Electrodeposition of FeCo-MOF on Au Ultramicroelectrode for Highly Sensitive Detection of Epinephrine. J. Electrochem..

[34] Araújo-Cordero A.M., Caddeo F., Mahmoudi B., Bron M., Maijenburg A.W. (2024). Direct Electrochemical Synthesis of Metal-Organic Frameworks: Cu_3_(BTC)_2_ and Cu(TCPP) on Copper Thin films and Copper-Based Microstructures. ChemPlusChem.

[35] Varsha M.V., Nageswaran G. (2020). Review—Direct Electrochemical Synthesis of Metal Organic Frameworks. J. Electrochem. Soc..

[36] Li W.-J., Tu M., Cao R., Fischer R.A. (2016). Metal–organic framework thin films: Electrochemical fabrication techniques and corresponding applications & perspectives. J. Mater. Chem. A.

[37] Zhang X., Wan K., Subramanian P., Xu M., Luo J., Fransaer J. (2020). Electrochemical deposition of metal–organic framework films and their applications. J. Mater. Chem. A.

[38] Xie S., Zhang X., Tan X., Zhang W., Guo W., Han N., Zhou Z., Jiang Y., Vankelecom I.F.J., Fransaer J. (2022). One-Step Reductive Electrodeposition of MOF Film on Polymer Membrane. ACS Mater. Lett..

[39] Usman M., Yang A.-C., Inamdar A.I., Kamal S., Hsu J.-C., Kang D.-Y., Tseng T.-W., Hung C.-H., Lu K.-L. (2022). Thin Film Growth of 3D Sr-based Metal-Organic Framework on Conductive Glass via Electrochemical Deposition. ChemistryOpen.

[40] Ji Y., Song Y., Huang Y., Zhu H., Yue C., Liu F., Zhao J. (2022). One-Step Synthesis of Ultrathin Zeolitic Imidazole Framework-8 (ZIF-8) Membrane on Unmodified Porous Support via Electrophoretic Deposition. Membranes.

[41] Ji H., Hwang S., Kim K., Kim C., Jeong N.C. (2016). Direct in Situ Conversion of Metals into Metal–Organic Frameworks: A Strategy for the Rapid Growth of MOF Films on Metal Substrates. ACS Appl. Mater. Interfaces.

[42] Shao M., Li J., Li J., Yan Y., Li R. (2023). Synthesis of Ni_3_S_2_ and MOF-Derived Ni(OH)_2_ Composite Electrode Materials on Ni Foam for High-Performance Supercapacitors. Nanomaterials.

[43] Shen W., Guo X., Pang H. (2022). Effect of Solvothermal Temperature on Morphology and Supercapacitor Performance of Ni-MOF. Molecules.

[44] Ravipati M., Badhulika S. (2023). Solvothermal synthesis of hybrid nanoarchitectonics nickel-metal organic framework modified nickel foam as a bifunctional electrocatalyst for direct urea and nitrate fuel cell. Adv. Powder Technol..

[45] Han G., Zhao S., Sui S., Feng B., Wang Y., Zhou J., Li Z., Tian X., Jia Y., Wang J. (2025). A New Type Microwave Absorption Materials: Metal–Organic-Frameworks (MOFs) Used as Non-Conductive Material Combination with P-C-800. Adv. Electron. Mater..

[46] Li J., Kumar A., Ott S. (2024). Diffusional Electron Transport Coupled to Thermodynamically Driven Electron Transfers in Redox-Conductive Multivariate Metal–Organic Frameworks. J. Am. Chem. Soc..

[47] Bhoite A.A., Patil K.V., Redekar R.S., Patil P.S., Sawant V.A., Tarwal N.L. (2023). Solvothermal synthesis of binder free Ni-MOF thin films for supercapacitor electrodes. J. Solid State Chem..

[48] Bhoite A.A., Sawant V.A., Tarwal N.L. (2024). Solvothermal synthesis of Ni/Co-based metal-organic framework with nanosheets-like structure for high-performance supercapacitor. Colloids Surf. A Physicochem. Eng. Asp..

[49] Suremann N.F., Laporte A.A.H., Greenwell F., Ott S. (2025). Pseudomorphic replication as enabling technology for porphyrinic metal–organic framework thin film growth. Polyhedron.

[50] Zheng J., Chen L., Kuang Y., Ouyang G. (2024). Universal Strategy for Metal-Organic Framework Growth: From Cascading-Functional Films to MOF-on-MOFs. Small.

[51] So M.C., Jin S., Son H.-J., Wiederrecht G.P., Farha O.K., Hupp J.T. (2013). Layer-by-Layer Fabrication of Oriented Porous Thin Films Based on Porphyrin-Containing Metal–Organic Frameworks. J. Am. Chem. Soc..

[52] Shekhah O., Wang H., Kowarik S., Schreiber F., Paulus M., Tolan M., Sternemann C., Evers F., Zacher D., Fischer R.A. (2007). Step-by-Step Route for the Synthesis of Metal−Organic Frameworks. J. Am. Chem. Soc..

[53] Arslan H.K., Shekhah O., Wieland D.C.F., Paulus M., Sternemann C., Schroer M.A., Tiemeyer S., Tolan M., Fischer R.A., Wöll C. (2011). Intercalation in Layered Metal–Organic Frameworks: Reversible Inclusion of an Extended π-System. J. Am. Chem. Soc..

[54] Arslan H.K., Shekhah O., Wohlgemuth J., Franzreb M., Fischer R.A., Wöll C. (2011). High-Throughput Fabrication of Uniform and Homogenous MOF Coatings. Adv. Funct. Mater..

[55] Chernikova V., Shekhah O., Eddaoudi M. (2016). Advanced Fabrication Method for the Preparation of MOF Thin Films: Liquid-Phase Epitaxy Approach Meets Spin Coating Method. ACS Appl. Mater. Interfaces.

[56] Li M.-H., Yang Z., Li Z., Wu J.-R., Yang B., Yang Y.-W. (2022). Construction of Hydrazone-Linked Macrocycle-Enriched Covalent Organic Frameworks for Highly Efficient Photocatalysis. Chem. Mater..

[57] Ma Y., Dong Z., You M., Zhang Y., Feng X., Ma X., Meng J. (2019). Formation of a thin and continuous MOF membrane with 2-D MOF nanosheets as seeds via layer-by-layer growth. Chem. Commun..

[58] Gao J., Wei W., Yin Y., Liu M., Zheng C., Zhang Y., Deng P. (2020). Continuous ultrathin UiO-66-NH2 coatings on a polymeric substrate synthesized by a layer-by-layer method: A kind of promising membrane for oil–water separation. Nanoscale.

[59] Chen D.-H., Haldar R., Neumeier B.L., Fu Z.-H., Feldmann C., Wöll C., Redel E. (2019). Tunable Emission in Heteroepitaxial Ln-SURMOFs. Adv. Funct. Mater..

[60] Fischer J.C., Steentjes R., Chen D.-H., Richards B.S., Zojer E., Wöll C., Howard I.A. (2024). Determining Structures of Layer-by-Layer Spin-Coated Zinc Dicarboxylate-Based Metal-Organic Thin Films. Chem. A Eur. J..

[61] Fratschko M., Zhao T., Fischer J.C., Werzer O., Gasser F., Howard I.A., Resel R. (2024). Thin Film Formation Based on a Nanoporous Metal–Organic Framework by Layer-By-Layer Deposition. ACS Appl. Nano Mater..

[62] Liu J., Chen Y., Huang X., Ren Y., Hambsch M., Bodesheim D., Pohl D., Li X., Deconinck M., Zhang B. (2024). On-liquid-gallium surface synthesis of ultra-smooth conductive metal-organic framework thin films. arXiv.

[63] Sindhu P., Ananthram K.S., Jain A., Tarafder K., Ballav N. (2022). Charge-transfer interface of insulating metal-organic frameworks with metallic conduction. Nat. Commun..

[64] Goswami S., Rimoldi M., Anderson R., Lee C., Li X., Li A., Deria P., Chen L.X., Schaller R.D., Gómez-Gualdrón D.A. (2022). Toward Ideal Metal–Organic Framework Thin-Film Growth via Automated Layer-by-Layer Deposition: Examples Based on Perylene Diimide Linkers. Chem. Mater..

[65] Barr M.K.S., Nadiri S., Chen D.-H., Weidler P.G., Bochmann S., Baumgart H., Bachmann J., Redel E. (2022). Solution Atomic Layer Deposition of Smooth, Continuous, Crystalline Metal–Organic Framework Thin Films. Chem. Mater..

[66] Romero-Ángel M., Rubio-Giménez V., Gómez-Oliveira E.P., Verstreken M.F.K., Smets J., Gándara-Loe J., Padial M.N., Ameloot R., Tatay S., Martí-Gastaldo C. (2023). Vapor-Assisted Conversion of Heterobimetallic Titanium–Organic Framework Thin Films. Chem. Mater..

[67] Xue L., Luo G., Yang X.-C., Qin Y., Zhang B. (2024). Vapor-phase methods for synthesizing metal-organic framework thin films. Innov. Mater..

[68] Wang B., Liu J., Mao C., Wang F., Yuan S., Wang X., Hu Z. (2024). A MOF-Gel Based Separator for Suppressing Redox Mediator Shuttling in Li–O_2_ Batteries. Small.

[69] Park C., Woo J., Jeon M., Baek J.W., Shin E., Kim J., Park S., Kim I.-D. (2025). Dual-MOF-Layered Films via Solution Shearing Approach: A Versatile Platform for Tunable Chemiresistive Sensors. ACS Nano.

[70] Zha X., Xi R., Wu Y., Xu J., Yang Y. (2022). Synthesis of Good Electrical Conductivity of CoCe-BTC/PEDOT for Ultrahigh Selectivity of NO_2_ Detection. Sensors.

[71] Zhestkij N.A., Povarov S.A., Volodin L., Chelmodeev R., Melkomukov M., Kenzhebayeva Y., Rzhevskiy S.S., Shipilovskikh S.A., Lubimova A.V., Timofeeva M.V. (2025). One-step flashlight processing of MOF thin films for non-linear light absorption. Mater. Chem. Front..

[72] Yang H., Giri A., Moon S., Shin S., Myoung J.-M., Jeong U. (2017). Highly Scalable Synthesis of MoS_2_ Thin Films with Precise Thickness Control via Polymer-Assisted Deposition. Chem. Mater..

[73] Abudayyeh A.M., Mahmoud L.A.M., Ting V.P., Nayak S. (2024). Metal–Organic Frameworks (MOFs) and Their Composites for Oil/Water Separation. ACS Omega.

[74] Mondal S.K., Aina P.O., Rownaghi A.A., Rezaei F. (2024). Design and development of UiO-67-coated PIM-1-based composites and demonstration of their detoxification performance. Chem. Eng. J..

[75] Gonzalez-Juarez M.d.L., Isaacs M.A., Bradshaw D., Nandhakumar I. (2023). Enhanced Thermoelectric Properties of a Semiconducting Two-Dimensional Metal–Organic Framework via Iodine Loading. ACS Appl. Mater. Interfaces.

[76] Yao M.-S., Lv X.-J., Fu Z.-H., Li W.-H., Deng W.-H., Wu G.-D., Xu G. (2017). Layer-by-Layer Assembled Conductive Metal–Organic Framework Nanofilms for Room-Temperature Chemiresistive Sensing. Angew. Chem. Int. Ed..

[77] Mandal P., Singh V., Zhang J., Tiwari M.K. (2025). Intercalated MOF nanocomposites: Robust, fluorine-free and waterborne amphiphobic coatings. Environ. Sci. Nano.

[78] Yang X.-X., Li C., Chen S.-M., Gu Z.-G., Zhang J. (2024). Layer by Layer Spraying Fabrication of Aggregation-Induced Emission Metal-Organic Frameworks Thin Film. Chem. A Eur. J..

[79] Zheng R., Fu Z.-H., Deng W.-H., Wen Y., Wu A.-Q., Ye X.-L., Xu G. (2022). The Growth Mechanism of a Conductive MOF Thin Film in Spray-based Layer-by-layer Liquid Phase Epitaxy. Angew. Chem. Int. Ed..

[80] Patil S.A., Katkar P.K., Kaseem M., Nazir G., Lee S.-W., Patil H., Kim H., Magotra V.K., Thi H.B., Im H. (2023). Cu@Fe-Redox Capacitive-Based Metal–Organic Framework Film for a High-Performance Supercapacitor Electrode. Nanomaterials.

[81] Mu A.U., Cai G., Chen Z. (2024). Metal–Organic Frameworks for the Enhancement of Lithium-Based Batteries: A Mini Review on Emerging Functional Designs. Adv. Sci..

[82] Xu Y., Zhao R., Fang J., Liang Z., Gao L., Bian J., Zhu J., Zhao Y. (2022). Metal-organic framework (MOF)-incorporated polymeric electrolyte realizing fast lithium-ion transportation with high Li^+^ transference number for solid-state batteries. Front. Chem..

[83] Saleem M., Ahmad F., Fatima M., Shahzad A., Javed M.S., Atiq S., Khan M.A., Danish M., Munir O., Arif S.M.B. (2024). Exploring new frontiers in supercapacitor electrodes through MOF advancements. J. Energy Storage.

[84] Jia H., Lu S., Shin S.H.R., Sushko M.L., Tao X., Hummel M., Thallapally P.K., Liu J., Gu Z. (2022). In situ anodic electrodeposition of two-dimensional conductive metal-organic framework@nickel foam for high-performance flexible supercapacitor. J. Power Sources.

[85] Salunkhe A.D., Pawar P.S., Pagare P.K., Torane A.P. (2024). Facile solvothermal synthesis of Ni-Co MOF/rGO nanoflakes for high-performance asymmetric supercapacitor. Electrochim. Acta.

[86] Islam R., Tan S., Afroj S., Karim N. (2024). Metal-Organic Framework (MOF)-based Smart E-textile Supercapacitors. ChemRxiv.

[87] Li X., Gao X., Gai P., Liu X., Li F. (2020). Degradable metal-organic framework/methylene blue composites-based homogeneous electrochemical strategy for pesticide assay. Sens. Actuators B Chem..

[88] Janjani P., Bhardwaj U., Gupta R., Singh Kushwaha H. (2022). Bimetallic Mn/Fe MOF modified screen-printed electrodes for non-enzymatic electrochemical sensing of organophosphate. Anal. Chim. Acta.

[89] De Chiara B., Del Duca F., Hussain M.Z., Kratky T., Banerjee P., Dummert S.V., Khoshouei A., Chanut N., Peng H., Al Boustani G. (2025). Laser-Induced Metal–Organic Framework-Derived Flexible Electrodes for Electrochemical Sensing. ACS Appl. Mater. Interfaces.

[90] Zhang S., Wang M., Wang X., Song J., Yang X. (2024). Electrocatalysis in MOF Films for Flexible Electrochemical Sensing: A Comprehensive Review. Biosensors.

[91] Wang X., Xu X., Zhou T., Zhang T. (2024). Nanoscale MOF-74-based QCM gas sensor for CO_2_ detection at room temperature. Sens. Actuators B Chem..

[92] Henkelis S.E., Percival S.J., Small L.J., Rademacher D.X., Nenoff T.M. (2021). Continuous MOF Membrane-Based Sensors via Functionalization of Interdigitated Electrodes. Membranes.

[93] Dai W., Wen K., Meng X., Yu S., Zhao J., Lin Q. (2024). Aptameric Metal–Organic Framework Nanobiosensor. ACS Appl. Nano Mater..

[94] Ke X., Zhao Z., Huang J., Liu C., Huang G., Tan J., Zhu H., Xiao Z., Liu X., Mei Y. (2023). Growth Control of Metal–Organic Framework Films on Marine Biological Carbon and Their Potential-Dependent Dopamine Sensing. ACS Appl. Mater. Interfaces.

[95] Majidi R., Farhadi A., Danaee I., Panah N.B., Zarei D., Nikmanesh S. (2023). Investigation of synthesized planar Cu-MOF and spherical Ni-MOF nanofillers for improving the anti-corrosion performance of epoxy coatings. Prog. Org. Coat..

[96] Hsia H.-H., Chen Y.-L., Tai Y.-T., Tian H.-K., Kung C.-W., Liu W.-R. (2024). Two-Dimensional Metal–Organic Frameworks/Epoxy Composite Coatings with Superior O_2_/H_2_O Resistance for Anticorrosion Applications. ACS Appl. Mater. Interfaces.

[97] Damian-Buda A.-I., Alipanah N., Bider F., Sisman O., Neščáková Z., Boccaccini A.R. (2025). Metal-organic framework (MOF)-bioactive glass (BG) systems for biomedical applications—A review. Mater. Today Bio.

[98] Gupta A., Bhardwaj S.K., Sharma A.L., Kim K.-H., Deep A. (2019). Development of an advanced electrochemical biosensing platform for *E. coli* using hybrid metal-organic framework/polyaniline composite. Environ. Res..

[99] Domke A., Jakubowski M., Ławniczak Ł., Ratajczak M., Voelkel A., Sandomierski M. (2024). Modification of titanium implants by bioactive and antibacterial zinc gallate metal organic framework. Surf. Coat. Technol..

[100] Chen K., Wang Y., Tang H., Niu X., Yang H., Bai Y., Gu X., Zheng Y. (2024). Fabrication of a Nanoscale Magnesium/Copper Metal–Organic Framework on Zn-Based Guided Bone Generation Membranes for Enhancing Osteogenesis, Angiogenesis, and Bacteriostasis Properties. ACS Appl. Mater. Interfaces.

[101] Nadeem T.B., Imran M., Tandis E. (2025). Applications of MOF-Based Nanocomposites in Heat Exchangers: Innovations, Challenges, and Future Directions. Nanomaterials.

[102] Ge L., Feng Y., Wu J., Wang R., Ge T. (2024). Performance evaluation of MIL-101(Cr) based desiccant-coated heat exchangers for efficient dehumidification. Energy.

[103] Fan Y., Liu Z., Chen G. (2022). Constructing flexible metal-organic framework/polymer/carbon nanotubes ternary composite films with enhanced thermoelectric properties for heat-to-electricity conversion. Compos. Commun..

[104] Kim H.K., Yun W.S., Kim M.-B., Kim J.Y., Bae Y.-S., Lee J., Jeong N.C. (2015). A Chemical Route to Activation of Open Metal Sites in the Copper-Based Metal–Organic Framework Materials HKUST-1 and Cu-MOF-2. J. Am. Chem. Soc..

[105] Peterson G.W., Lee D.T., Barton H.F., Epps T.H., Parsons G.N. (2021). Fibre-based composites from the integration of metal–organic frameworks and polymers. Nat. Rev. Mater..

[106] Abednatanzi S., Derakhshandeh P.G., Depauw H., Coudert F.-X., Vrielinck H., Van Der Voort P., Leus K. (2019). Mixed-metal metal–organic frameworks. Chem. Soc. Rev..

[107] Elrasheedy A., Nady N., Bassyouni M., El-Shazly A. (2019). Metal Organic Framework Based Polymer Mixed Matrix Membranes: Review on Applications in Water Purification. Membranes.

[108] Albdoor A.K., Ma Z., Cooper P., Al-Ghazzawi F., Liu J., Richardson C., Wagner P. (2021). Air-to-air enthalpy exchangers: Membrane modification using metal-organic frameworks, characterisation and performance assessment. J. Clean. Prod..

[109] Li H., Yan M., Zhao W. (2022). Designing a MOF-based slippery lubricant-infused porous surface with dual functional anti-fouling strategy. J. Colloid Interface Sci..

[110] Zhang L., Li H., Zhang X., Li Q., Zhang G., Pu S., Liu F.-Q. (2024). UV-curable PBMA coating containing CuZn-MOF-74 for fouling-resistance. Microporous Mesoporous Mater..

[111] Moaness M., El-Sayed S.A.M., Beherei H.H., Mabrouk M. (2024). Enhancing the Antifouling Properties of Alumina Nanoporous Membranes by GO/MOF Impregnated Polymer Coatings: In Vitro Studies. J. Funct. Biomater..

[112] Wang M., Zi Y., Zhu J., Huang W., Zhang Z., Zhang H. (2021). Construction of super-hydrophobic PDMS@MOF@Cu mesh for reduced drag, anti-fouling and self-cleaning towards marine vehicle applications. Chem. Eng. J..

[113] Chaemchuen S., Xiao X., Klomkliang N., Yusubov M.S., Verpoort F. (2018). Tunable Metal–Organic Frameworks for Heat Transformation Applications. Nanomaterials.

[114] Shahvari S.Z., Kalkhorani V.A., Clark J.D. (2022). Performance evaluation of a metal organic frameworks based combined dehumidification and indirect evaporative cooling system in different climates. Int. J. Refrig..

[115] Hou P., Zu K., Qin M., Cui S. (2021). A novel metal-organic frameworks based humidity pump for indoor moisture control. Build. Environ..

[116] Zu K., Qin M. (2022). Optimization of the hygrothermal performance of novel metal-organic framework (MOF) based humidity pump: A CFD approach. Energy.

[117] Kummer H., Jeremias F., Warlo A., Füldner G., Fröhlich D., Janiak C., Gläser R., Henninger S.K. (2017). A Functional Full-Scale Heat Exchanger Coated with Aluminum Fumarate Metal–Organic Framework for Adsorption Heat Transformation. Ind. Eng. Chem. Res..

[118] Aziz A.N., Mahmoud S., Al-Dadah R., Taskin A., Ismail M.A., Fahmy Y.M., Rashid M.M. (2024). Novel MOF-303/G coated wire-finned heat exchanger for dehumidification applications–Experimental investigation. Energy.

[119] Albaik I., Al-Dadah R., Mahmoud S., Solmaz İ. (2021). Non-equilibrium numerical modelling of finned tube heat exchanger for adsorption desalination/cooling system using segregated solution approach. Appl. Therm. Eng..

[120] Wang S., Fan Y., Wang Y., Gui L., Huang S., Wu T., Tian X. (2024). Light-Responsive Metal–Organic Framework (MOF)-Based Liquid-Like Coating for Sustainable Scale Resistance. Adv. Funct. Mater..

